# A new triazolothiadiazine derivative inhibits stemness and induces cell death in HCC by oxidative stress dependent JNK pathway activation

**DOI:** 10.1038/s41598-022-17444-0

**Published:** 2022-09-07

**Authors:** Deniz Cansen Kahraman, Ebru Bilget Guven, Peri S. Aytac, Gamze Aykut, Birsen Tozkoparan, Rengul Cetin Atalay

**Affiliations:** 1 Cancer Systems Biology Laboratory, Graduate School of Informatics, METU, 06800 Ankara, Turkey; 2 Department of Molecular Biology and Genetics, Bilkent University, 06800 Ankara, Turkey; 3 Department of Molecular Biology and Genetics, Kadir Has University, 34083 Istanbul, Turkey; 4 Department of Pharmaceutical Chemistry, Hacettepe University, 06800 Ankara, Turkey; 5 Section of Pulmonary and Critical Care Medicine, the University of Chicago, Chicago, IL 60637 USA

**Keywords:** Cancer stem cells, Drug screening, Medicinal chemistry, Drug development, Cell signalling, Cell death, Apoptosis, Small molecules, Liver cancer

## Abstract

Hepatocellular carcinoma (HCC) is a highly heterogeneous cancer, and resistant to both conventional and targeted chemotherapy. Recently, nonsteroidal anti-inflammatory drugs (NSAIDs) have been shown to decrease the incidence and mortality of different types of cancers. Here, we investigated the cellular bioactivities of a series of triazolothiadiazine derivatives on HCC, which have been previously reported as potent analgesic/anti-inflammatory compounds. From the initially tested 32 triazolothiadiazine NSAID derivatives, 3 compounds were selected based on their IC_50_ values for further molecular assays on 9 different HCC cell lines. **7b,** which was the most potent compound, induced G2/M phase cell cycle arrest and apoptosis in HCC cells. Cell death was due to oxidative stress-induced JNK protein activation, which involved the dynamic involvement of ASK1, MKK7, and c-Jun proteins. Moreover, **7b** treated nude mice had a significantly decreased tumor volume and prolonged disease-free survival. **7b** also inhibited the migration of HCC cells and enrichment of liver cancer stem cells (LCSCs) alone or in combination with sorafenib. With its ability to act on proliferation, stemness and the migration of HCC cells, **7b** can be considered for the therapeutics of HCC, which has an increased incidence rate of ~ 3% annually.

## Introduction

Hepatocellular carcinoma (HCC) is one of the most common cancer types and second in terms of cancer-related mortality worldwide^1^. HCC has a very heterogeneous structure and develops over many years as a multi-step process^2^. Patients with advanced stage HCC have very limited and mostly palliative treatment options due to the chemo-resistant nature of the disease^3,4^. Hepatic injury initiated by any of the etiologies such as chronic hepatitis, alcohol abuse or aflatoxin-B1-intoxication, and recently obesity, results in continuous destructive-regenerative cycles and causes cirrhosis, which further induces carcinogenesis leading to HCC^5^. Recently, sorafenib, lenvatinib, regorafenib, atezolizumab plus bevacizumab, cabozantinib and ramucirumab are the FDA-approved drugs for the systemic therapy of HCC^6^. Although sorafenib and Lenvatinib are regarded as the best treatment options for advanced HCC patients, capable of inducing apoptosis and inhibiting angiogenesis and proliferation of tumor cells, they can extend patient survival for about 3 months^7,8^. Combinatorial approaches involving the use of chemotherapeutic agents together with sorafenib in HCC have been reported to show limited usage due to the high toxicity and unconvincing efficacy in prolonging the survival of HCC patients^9,10^. In addition, recent studies report that acquired drug resistance to sorafenib is very common. Multiple factors and mechanisms are identified to be involved in the development of sorafenib resistance followed by the progression of the disease. Tumor microenvironment, EGFR activation, compensatory pathways such as PI3K/AKT and JAK/STAT, and the presence of cancer stem cells (CSCs) are only some of these mechanisms^11^. Therefore, it is essential to discover reliable drug candidates that could provide novel therapeutic options for advanced HCC patients.

**Figure 1:**
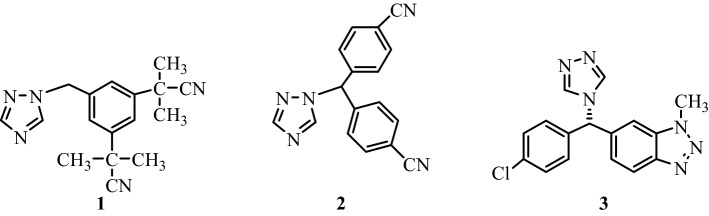
Structures of anastrozole, letrozole, and vorozole used in treatment of estrogen receptor positive breast cancer as nonsteroidal aromatase inhibitors.

Nonsteroidal anti-inflammatory drugs (NSAIDs) have been used to treat acute and chronic conditions with pain and inflammation for many years. Recently, clinical studies have emphasized the role of NSAIDs in cancer treatment, such that NSAIDs are effective in decreasing the incidence and mortality of many cancer types^12–15^. 1,2,4-Triazole is an important scaffold in the field of medicinal chemistry. Currently, a group of compounds carrying the 1,2,4-triazole ring, named anastrozole (1), letrozole (2) vorozole (3), are explicitly used in the treatment of estrogen receptor-positive breast cancer as nonsteroidal aromatase inhibitors (Fig. 1)^16,17^. For this reason, the five-membered 1,2,4-triazole ring system is a suitable heterocyclic core structure for designing new anticancer compounds. During the last two decades, it has been determined that triazolopyridazine, triazolotriazine, triazolothiadiazine, and triazolothiadiazole derivatives (condensed 1,2,4-triazole derivatives) have cytotoxic activities in many cancer cells by inducing various molecular mechanisms^18–25^.

**Figure 2:**
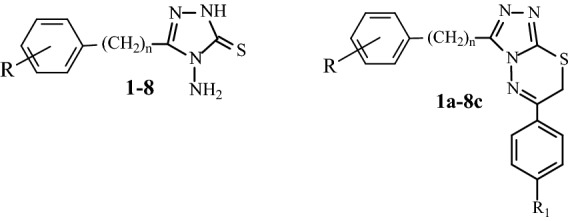
The chemical structure of the compounds involved in this study.

Since more than 90% of the liver cancer cases are associated with chronic inflammation^26^, NSAIDs could be effective against HCC. Indeed, in one of our previous studies, the anticancer effects of triazolothiadiazines on epithelial cancers, especially liver cancer, were demonstrated, where compounds could induce apoptotic cell death due to oxidative stress, acting on Akt protein^13^. Furthermore, a compound with triazolothiadiazine scaffold, displaying antiproliferative bioactivities against human hepatoma cell line (HepG2), was reported recently^27^. In light of this information and as a part of our ongoing interest in liver cancer therapeutics^28^, we investigated the anticancer effects of our newly synthesized compounds bearing triazolethiones (**1–8**) or triazolothiadiazine (**1a-8c**) cores, which we reported as effective analgesic/anti-inflammatory compounds previously (Fig. 2)^24,25^.

This study not only evaluates the in vitro and in vivo anti-tumor activity of the compounds against liver cancer, but also characterizes the form of cell death induced by the compounds and identifies the underlying molecular mechanism of action with the most potent compound.

## Materials and methods

### Synthesis of NSAID triazolothiadiazine derivatives

Synthesis of 4-amino-3-substituted-1,2,4-triazole-5-thiones (Compounds **1–8**), was carried out by melting some aralkyl carboxylic acid derivatives with thiocarbohydrazide according to the procedure described previously^25,29^. Synthesis of 3,6-disubstituted-7*H*-1,2,4-triazolo[3,4-*b*]-1,3,4-thiadiazines (**1a-8c**) was carried out by reacting relevant 4-amino-3-substituted-1,2,4-triazole-5-thiones (**1–8**) with appropriate phenacyl halides in anhydrous ethanol under reflux, 3,6-disubstituted-7*H*-1,2,4-triazolo[3,4-*b*]-1,3,4-thiadiazines (**1a-8c**)^25,29^. Physical properties and spectral data characterizing the structure of the synthesized compounds were reported previously^24,25^.

### Drugs and Chemicals

JNK inhibitor V (cat. no. 420129) and DMSO (cat. no. D2650) were provided from Calbiochem;Sigma-Aldrich; Merck KGaA (Burlington, MA, United States), sorafenib (cat. no. S7397), and DAPT (cat. no. S2215) were provided from Selleck chemicals (Houston, TX, USA), Taxol was provided from Bristol Myers Squibb (New York City, New York, USA). Camptothecin (cat. no. C9911) was purchased from Sigma-Aldrich; Merck KGaA. N-acetyl-cysteine (NAC) was provided from Birsen Tozkoparan (Department of Pharmaceutical Chemistry, Hacettepe University, Ankara, Turkey).

### Cell culture

Nine hepatocellular carcinoma, and one colon carcinoma cell lines were obtained from the following sources: Huh7 (JCRB0403), HepG2 (ATCC HB-8065), Hep3B (ATCC HB-8064), PLC (ATCC CRL-8024), SK-Hep1 (ATCC HTB52) Mahlavu^30^, FOCUS^31^, SNU182 (ATCC CRL-2235), SNU387 (ATCC CRL-2237), SNU475 (ATCC CRL-2236) and, HCT116 (ATCC CCL-247). Cells were grown in DMEM (cat. no. 31885), (Dulbecco's Modified Eagle Medium) or RPMI (Roswell Park Memorial Institute)-1640 growth medium (cat. no. 31870) supplemented with 10% fetal bovine serum (FBS) (cat. no. 10270), 1% non-essential amino acids (cat. no. 11140050), 1% L-glutamine (cat. no. 25030), 1% penicillin and streptomycin (cat. no. 15140–122) from Invitrogen; Thermo Fisher Scientific, Inc. (Waltham, MA, USA) at 37 °C under 5% CO_2_. All cell lines used in this study are STR (short tandem repeat) authenticated. Cells are regularly tested for mycoplasma contamination using a mycoplasma detection kit (cat. no. LT07-318) (MycoAlert™, Lonza, Basel, Switzerland). The passaging of the cells did not exceed 8–10 passages (2 times a week) throughout the experiments.

### Sulforhodamine B (SRB) assay

Cells were inoculated into 96-well plates (1000–5000 cell/well in 150 μL/well) for 24 h and treated with the increasing concentrations of compounds (40–0.01 µM) for 24, 48, or 72 h. Cells were washed with 1xPBS (CaCl2-, MgCl2-free) (Invitrogen, cat. no. 14190144) and fixed with ice-cold 10% trichloroacetic acid (cat. no. 27242, Sigma Aldrich; Merck KGaA,). Microplates were kept at + 4 °C, in the dark for 1 h, then washed with ddH2O. Air-dried plates were stained with SRB (cat. no. 2S1403 Sigma Aldrich; Merck KGaA) (w/v 0.4%SRB in 1% acetic acid solution) for 10 min in the dark. The plates were rinsed with 1% acetic acid solution to wash away the unbound SRB. The protein-bound SRB was then solubilized in a 10 mM Tris-base solution. The absorbance values were obtained at 515 nm using a ELx800 plate reader (BioTek Instruments, Inc., Winooski, VT, USA).

### Real-time cell growth analysis (RT-CES)

HCC cells were plated into E-96 plates (1000–5000 cells/well). The cell index (CI) values were recorded to obtain the proliferation curve of the cells through real-time cell electronic sensing (RT-CES) (ACEA Biosciences; Agilent Technologies, Santa Clara, CA, USA). On the next day, the cells were treated with the compounds. The CI values, representing the cell impedance, were recorded every 10 min for the first 4 h to monitor the fast drug response and then every 30 min to monitor the long-term drug response. Cell growth curves were generated using the time-zero normalized CI values.

### Annexin V/PI staining analysis of apoptosis

Cells were plated into 6-well plates (30,000–60,000 cells/well). The next day cells were treated in increasing concentrations of **7b** or DMSO. For experiments with NAC and JNKinhV, cells were treated with either NAC (5 mM), JNKinhV (1 µM) only, or pre-treated with NAC for 1 h followed by **7b** treatment, or with JNKinhV and **7b** (simultaneously) for 24 h. Cells were harvested in falcon tubes by trypsinization and centrifuged at 1500 rpm for 5 min. Cell media was removed, and pellets were washed twice in ice-cold 1xPBS and centrifuged again. Then, cells were resuspended in Annexin-V-FLUOS labeling solution which consisted of 2 µl Annexin-V-FLUOS labeling reagent and 2 µL PI solution in 100 µl incubation buffer per sample and incubated for 15 min at room temperature in dark. 100–200 µl of incubation buffer was added on top of each sample and analyzed using Novocyte (ACEA Biosciences; Agilent Technologies) flow cytometry. The percentage of apoptotic cells was determined using NovoExpress software (ACEA Biosciences; Agilent Technologies).

### Cell cycle analysis

HCC cells were inoculated in 100 mm culture dishes. After 24 h, cells were treated with compound **7b** and its DMSO control for 24 h. For experiments with NAC, cells were treated with either NAC (5 mM) or pre-treated with NAC for 1 h followed by **7b** treatment for 24 h. Cell pellets were harvested and re-suspended in 1 ml, ice-cold 1xPBS, and fixed by adding 2.5 mL, 70% ice-cold ethanol. Next, the cell pellets were re-suspended in Propidium iodide (PI) solution (50 μg/mL PI (cat. no. P4854, Sigma Aldrich; Merck KGaA), 0.1 μg/mL RNaseA (cat. no. EN0531Fermentas;Thermo Fisher Scientific Inc.), 0.05% Triton-X-100, 1xPBS) and incubated for 40 min at 37 °C at dark. Cell cycle analysis was performed using FACSCalibur and CellQuest Software (BD Biosciences, Franklin Lakes, NJ, USA) or Novocyte Flow Cytometry (ACEA Biosciences; Agilent Technologies).

### Detection of ER-stress

Total RNA was isolated from cells via the Nucleospin RNA II kit (cat. no. 740955, Macherey–Nagel, Düren, Germany) according to the manufacturer’s protocol. First strand cDNA synthesis was performed using the RevertAid First Strand cDNA synthesis kit (cat. no. K1622, Thermo Scientific Inc.). Semi-quantitative reverse transcriptase PCR (RT-PCR) assay was performed using XBP1 specific primers. The primer sequences; GAPDH forward primer: GGCTGAGAACGGGAAGCTTGTCAT, GAPDH reverse primer: CAGCCTTCTCCATGGTGGTGAAGA, XBP1 forward primer: TTACGAGAGAAAACTCATGGCC, XBP1 reverse primer: GGGTCCAAGTTGTCCAGAATGC.

### Reactive Oxygen Species (ROS) detection

Huh7 and Mahlavu cells were seeded into 10 cm culture dishes (100,000–300,000 cells/dish). Next day, cells were treated with compound **7b**, DMSO or Selenium (Se) deficient serum-free media for 8, 12 or 24 h. For experiments with NAC, cells were treated with compound **7b** only or pretreated with NAC for 1 h following **7b** treatment for 12 h. Flow cytometric analysis of ROS accumulation in cells at 12 h was done with the Oxidative Stress kit (cat. no. MCH100111, Merck Millipore) using the MUSE™ Cell Analyzer. In parallel, cells were incubated with ROS assay solution (10 mM HEPES buffer (cat. no. H-1016, Sigma Aldrich; Merck KGaA) 10 mM glucose (cat. no 0.0188, Amresco, OH, USA), 1 µM DCFH-DA (Dichloro-dihydro-fluorescein diacetate) (cat. no. D6883, Sigma Aldrich; Merck KGaA) in 1 × PBS) for 15 min at 37 °C and then visualized under a fluorescence microscope (Nikon Eclipse Ti-E, Minato City, Tokyo, Japan). Se-deficient serum-free medium was used as the positive control ^34^.

### Western Blot analysis

HCC cells were treated with the increasing concentrations of compound **7b** (+ : IC_50_, +  + : 2 × IC_50_) or with DMSO control. For experiments with NAC, cells were treated with either NAC (5 mM) or pre-treated with NAC for 1 h followed by **7b** treatment for 24 h. Cells were harvested by scraping, washed with PBS, lysed with RIPA lysis buffer on ice and centrifuged at 13,000 rpm for 20 min. The supernatants were collected, and the protein concentration was measured using the Bradford assay. For SDS-PAGE, 20–50 µg of protein was prepared, and samples were run using the Novex® NuPAGE® Bis–Tris Electrophoresis system (Thermo Fisher Scientific Inc.) according to the manufacturer’s protocol. Transfer of proteins to nitrocellulose membrane was done via XCell IITM Blot Module (Thermo Fisher Scientific Inc.). The blots were incubated with primary antibodies against PARP-1 (Santa Cruz Biotechnology, Dallas, TX, USA, cat. no. sc‐8007, 1:1,000), caspase-3 (Cell Signaling Technology, Danvers, MA, USA, cat no. 9661S, 1:200), caspase-9 (Santa Cruz Biotechnology, cat no. sc-22182, 1:200), p53 (Cell Signaling Technology cat. no. 9286S, 1:1000), p21 (Cell Signaling Technology cat. no. 2946S), cyclin B1 (Cell Signaling Technology cat. no. 4135S), CDK1, SAPK/JNK (Cell Signaling Technology cat no. 9252, 1:300), Phospho-SAPK/JNK (Thr183/Tyr185) (Cell Signaling Technology cat. no. 9251, 1:200), phospho-p38 (Thr180/Tyr182) (Cell Signaling Technology cat. no. 9211S, 1:500), phospho-c-Jun (Santa Cruz Biotechnology cat. no. sc-822, 1:200), phospho-ASK1 (Thr845) (Cell Signaling Technology cat. no. 3765, 1:500), phospho-ASK1 (Ser966) (Genscript, Piscataway, NJ, USA, cat. no. A00340, 1:500), phospho-ASK1 (Ser83) (Abcam, Cambridge, UK, cat. no. ab47304, 1:500), phospho-MKK7 (Ser271/Thr275) (Cell Signaling Technology cat. no. 4171, 1:500), phospho-MKK4 (S257 + T261) (Abcam cat. no. ab4760, 1:300), calnexin (Sigma-Aldrich; Merck KGaA cat. no. C4731, 1:5000), and actin (Santa Cruz Biotechnology cat. no. sc-1616, 1:5000) in 0.1% TBST at 4 °C overnight, followed by secondary antibody incubations with HRP-conjugated goat anti-mouse IgG (Sigma-Aldrich; Merck KGaA cat. no. A0168, 1:5000), rabbit anti-goat IgG (Sigma-Aldrich; Merck KGaA cat. no. A8919, 1:5000), or goat anti-rabbit IgG (Sigma-Aldrich; Merck KGaA cat. no. A6154, 1:5000) for 1 h at room temperature. Proteins were visualized by using the enhanced chemiluminescence (ECL) system or C-DiGit Blot Scanner (LI-COR, Lincoln, NE, USA). All original western blot images are provided in Supplementary Fig. S8.

### In vivo mouse xenograft experiments

All animals received human care, and study protocols comply with the institution's guidelines. The animal ethics committee of Bilkent University approved the study protocol. In addition, all studies were reported in accordance with the ARRIVE (Animal Research: Reporting of In Vivo Experiments) guidelines. Mahlavu cells prepared in DMEM (10,000,000 cells/mouse) were injected subcutaneously (SC) to the flank of 8–16 weeks old male nude mice as described previously^35^. Drug treatment was initiated once the tumor volume reached 150mm^3^. The subjects received compound **7b** (100 mg/kg) suspended in simple syrup (16 g glucose in 9 g ddH_2_O) by oral feeding, and the control group of mice received only 100 µl simple syrup twice a week for 21 days. Nude mice were not treated for the following 3 weeks and imaged with Magnetic Resonance Imaging (acquired with 3-TESLA Siemens MAGNETOM Trio, UMRAM Center, Bilkent University) following intraperitoneal (ip) injection of anesthesia regimen consisting of 10 mg/kg xylaxin and 90 mg/kg ketamine.

### Detection of liver cancer stem cell (LCSC) enrichment by flow cytometry

Huh7 and Mahlavu cells were seeded onto 100 mm culture dishes. The next day cells were treated with compound **7b**, sorafenib at their IC_50_ concentrations or their combinations, and corresponding DAPT (positive), or DMSO (vehicle) controls. Fluorescence labeling of LCSCs was done using primary antibodies against CD133 (Miltenyi Biotec, Bergisch Gladbach, North Rhine-Westphalia, Germany cat. no. 130-090-664), and anti-biotin-PE (Miltenyi Biotec, cat. no. 130-090-756), EpCAM (Miltenyi Biotec, cat. no. 130–080-301), or CD90 (Miltenyi Biotec, cat. no. 130-095-403) for flow cytometry analysis as described previously^36^. Mouse-IgG-FITC (Miltenyi Biotec, cat. no. 130–092-213), and mouse-IgG-biotin antibodies (Miltenyi Biotec, cat. no. 130-093-018) were used as isotype controls. DAPT (Notch pathway inhibitor) was used as a positive control for cancer stem cell inhibition. Results for each treatment group were compared to that of the DMSO control. Changes in the positivity of CD133 + /EpCAM + cells or CD90 + cells were indicative of enrichment or reduction of the LCSC population. BD Accuri C6 and Novoctye flow cytometer (ACEA Biosciences; Agilent Technologies) were used for flow cytometric analysis.

### Sphere formation assay

Sphere formation was triggered using DMEM/F12 serum-free medium (Invitrogen) supplemented with epidermal growth factor (20 ng ml^−1^; cat. no. PHG0311, Thermo Fisher Scientific Inc.), basic fibroblast growth factor (10 ng ml^−1^; cat. no. F0291, Sigma Aldrich; Merck KGaA), B27 supplement (1:50; cat. no. 10889038, Invitrogen; Thermo Fisher Scientific Inc.), Heparin (2 µg ml^−1^; cat. no. H3149, Sigma Aldrich; Merck KGaA), insulin (5 µg ml^−1^; cat. no. I-6634, Sigma Aldrich; Merck KGaA), hydrocortisone (0.5 µg ml^−1^; cat. no. H0888, Sigma Aldrich; Merck KGaA) and 100 units mL^-1^ penicillin and streptomycin (Invitrogen; Thermo Fisher Scientific Inc). The cells were cultured in ultra-low attachment 96-well plates (cat. no. CLS3474-24EA, Corning, Millipore; Merck KGaA) for 6–12 days. Images of spheres and measurements of sphere size and number were assessed using light microscopy (Zeiss, Oberkochen, Germany).

### Cell migration assay

RT-CES DP system (ACEA Biosciences; Agilent Technologies) was used to test the effect of compound **7b** on the migration capacity of Huh7 and Mahlavu cells. The lower chamber of the 16-well CIM-plate (cat. no. 5665817001, ACEA Biosciences; Agilent Technologies) was filled with 160 µl of 10% FBS containing complete DMEM, and the upper chamber was placed on top of the lower chamber. After 1 h at 37 °C, cells that were prepared in the presence of different concentrations of compound **7b** (2 µM for Huh7 and 3 µM for Mahlavu), Taxol (20 ng/ml) or DMSO inside serum-free DMEM, to be seeded into the upper chamber (30,000–50,000 cell/well). CIM-plates were placed into the system after 30 min of incubation at room temperature (RT), and CI values were obtained every 15 min for 24 h. Time-zero normalized CI values were used to generate time-dependent migration curves for each experimental group.

### Statistical analysis

Data were obtained from three independent experiments, and all experiments were carried out with n ≥ 3 biological replicates. Statistical analysis for in vitro data was done using a Student's t-test. All in vivo experimental data were analyzed using ANOVA, *n* = 5–6 mice/group (Graphpad Prism version 7.0, or Microsoft Excel). * *p* < 0.05, ** p < 0.01, *** *p* < 0.001.

## Results

### Cytotoxic bioactivities of 4-amino-3-substituted-1,2,4-triazole-5-thiones (1–8) and 3,6-disubstituted-7H-1,2,4-triazolo[3,4-b]-1,3,4-thiadiazines (1a-8c) on cancer cells

Cytotoxic bioactivities of 8 compounds with 4-amino-3-substituted-1,2,4-triazole-5-thiones (**1–8**) and 24 compounds with 3,6-disubstituted-7*H*-1,2,4-triazolo[3,4-*b*]-1,3,4-thiadiazines **(1a-8c)** were tested on primary liver and colon cancer cell lines by SRB assay (Table 1). While 4-amino-3-substituted-1,2,4-triazole-5-thiones (**1–8**) did not exhibit cytotoxic activities on two epithelial cancer cell lines (Huh7 and HCT116), compounds with triazolothiadiazine core had significant cytotoxicity, comparable to the effect of a well-known chemotherapeutic drug camptothecin (CPT). Among all the compounds, three of them (**7a, 7b, and 7c**) had prominent cytotoxic activities at concentrations ranging from 0.3–5.3 µM (Table 1). These compounds were then screened against a panel of HCC cell lines (Huh7, HepG2, Hep3B, PLC, SK-Hep1, Mahlavu, FOCUS, SNU182, and SNU475). IC_50_ values were ~ 7–23 µM for **7a**, ~ 0.2–1 µM for **7b** ~ 12–50 µM for **7c** (Supplementary Table S1). With cytotoxic doses smaller than 5 µM, compound **7b** was identified as a potent anticancer agent against liver cancer cells. Interestingly, this compound was previously reported to have the most promising and reliable anti-inflammatory activity among newly synthesized 3,6-disubstituted 7*H*-1,2,4-triazolo[3,4-b]-1,3,4-thiadiazine derivatives^25,29^. Next, we evaluated the anticancer activities of compound **7b** against a panel of HCC cell lines consisting of Huh7, HepG2, Mahlavu, FOCUS, SNU475, and SNU387 to determine the time- and dose-cell growth inhibitory effect. Time-dependent IC_50_ values of compound **7b** for each HCC cell line were calculated accordingly. (Table 2).

**Table 1:** IC_50_ values of 4-amino-3-substituted-1,2,4-triazole-5-thiones (**1–8**) and 3,6-disubstituted 1,2,4-triazolo[3,4-b]-1,3,4-thiadiazines (**1a-8c**) determined by SRB assay.

Compound	R	R_1_	*n*	Huh7	HCT116
**1**	2-OCH_3_	–	1	NI	NI
**1a**	2-OCH_3_	H	1	37.6	> 40
**1b**	2-OCH_3_	Cl	1	NI	NI
**1c**	2-OCH_3_	F	1	> 40	NI
**2**	2- OCH_3_	–	2	NI	NI
**2a**	2-OCH_3_	H	2	NI	NI
**2b**	2-OCH_3_	Cl	2	23	NI
**2c**	2-OCH_3_	F	2	> 40	NI
**3**	3- OCH_3_	–	1	NI	> 40
**3a**	3-OCH_3_	H	1	24.7	30.9
**3b**	3-OCH_3_	Cl	1	> 40	NI
**3c**	3-OCH_3_	F	1	> 40	NI
**4**	3- OCH_3_	–	2	NI	NI
**4a**	3-OCH_3_	H	2	> 40	NI
**4b**	3-OCH_3_	Cl	2	> 40	NI
**4c**	3-OCH_3_	F	2	NI	NI
**5**	4- OCH_3_	–	1	NI	> 40
**5a**	4-OCH_3_	H	1	> 40	> 40
**5b**	4-OCH_3_	Cl	1	> 40	NI
**5c**	4-OCH_3_	F	1	28.6	NI
**6**	4- OCH_3_	–	2	NI	> 40
**6a**	4-OCH_3_	H	2	18.8	35.4
**6b**	4-OCH_3_	Cl	2	30.1	> 40
**6c**	4-OCH_3_	F	2	NI	NI
**7**	3,4,5- OCH_3_	–	1	NI	NI
**7a**	3,4,5- OCH_3_	H	1	5	15
**7b**	3,4,5- OCH_3_	Cl	1	0.3	< 0.1
**7c**	3,4,5- OCH_3_	F	1	5.3	17.2
**8**	3,4,5- OCH_3_	–	2	NI	NI
**8a**	3,4,5- OCH_3_	H	2	27.8	NI
**8b**	3,4,5- OCH_3_	Cl	2	13.7	> 40
**8c**	3,4,5- OCH_3_	F	2	40	NI
CPT				< 0.1	< 0.1

*NI: no inhibition.

**Table 2:** IC_50_ values of compound **7b** in µM concentrations against Huh7, HepG2, Mahlavu, FOCUS, SNU475 and SNU387 cells at 24, 48 and 72 h determined by SRB assay.

	**24h**	**48h**	**72h**
Huh7	0.7 ± 0.20	0.2 ± 0.10	0.2 ± 0.01
HepG2	1.9 ± 0.80	1.1 ± 0.20	1.3 ± 0.30
Mahlavu	1.3 ± 0.30	1.2 ± 0.10	1.3 ± 0.20
FOCUS	0.8 ± 0.10	0.5 ± 0.10	0.4 ± 0.01
SNU475	2.1 ± 0.20	1.6 ± 0.20	1.7 ± 0.20
SNU387	4.7 ± 1.00	1.3 ± 0.20	2.5 ± 0.40

### Cell growth inhibition, apoptosis, and cell cycle arrest induction by compound 7b

**Figure 3:**
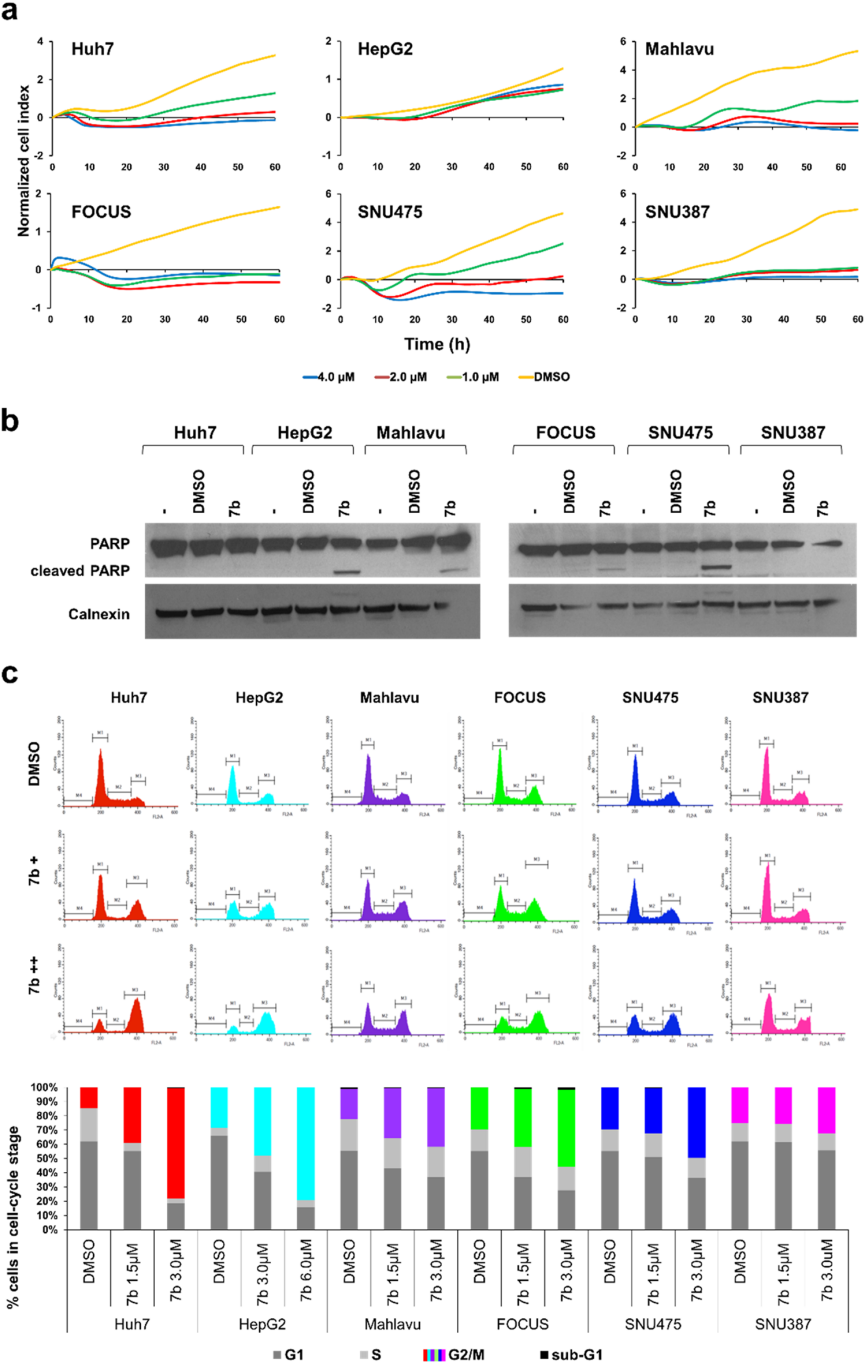
**7b** induces growth inhibition, apoptosis, and cell cycle arrest in HCC cell lines. (**a**) Real-time cell growth in the presence of compound **7b** and its DMSO control. The cell growth index of **7b** treated cells was recorded every 30 min. All compounds were administered in triplicates for RT-CES analysis**. **(**b**) Western blot analysis displaying the cleaved PARP upon compound **7b** treatment. All HCC cell lines were treated with 2.5 μM **7b** or its corresponding DMSO for 24 h. Calnexin protein was used as an equal loading control. (**c**) Histograms and the bar graph indicating cell cycle distribution of Huh7, HepG2, Mahlavu, FOCUS, SNU475 and SNU387 cells treated with increasing concentrations of compound **7b** and their DMSO controls for 24 h.

A label-free real-time cell monitoring system (RT-CES) that can measure cell growth by electrical impedance detection was used to determine the real-time anticancer activity of compound **7b**. It was demonstrated that compound **7b** inhibits cell growth in a dose- and cell line-dependent manner, similar to our initial results with the SRB assay (Fig. 3a). Further experiments were performed to decipher the mechanism underlying this activity. In the presence of compound **7b**, cleaved Poly-ADP-ribosyl-polymerase (PARP) fragments indicative of activated apoptotic pathway activation, were visible in most of the HCC cells after 24 h (Fig. 3b). The percentage of apoptotic cells increased significantly, as shown by Annexin-V/PI staining (Supplementary Fig. S1a). Furthermore, caspase-9 and caspase-3 cleavage was also observed, indicating activation of intrinsic apoptotic pathway upon **7b** treatment in HCC cells (Supplementary Fig. S1b). To further enlighten the mechanism beneath the apoptotic cell death in these cells, cell cycle distribution in the presence of compound **7b** was examined by flow cytometry analysis of propidium iodide-stained cells. Compound **7b** induced dose-dependent G2/M arrest in HCC cell lines after 24 h (Fig. 3c). In addition, **7b** caused a significant increase in p53 and p21 expression levels and a minor decrease in the levels of CDK1 in HCC cells (Supplementary Fig. S2).

### Induction of ROS accumulation and activation of JNK pathway upon treatment with 7b

**Figure 4:**
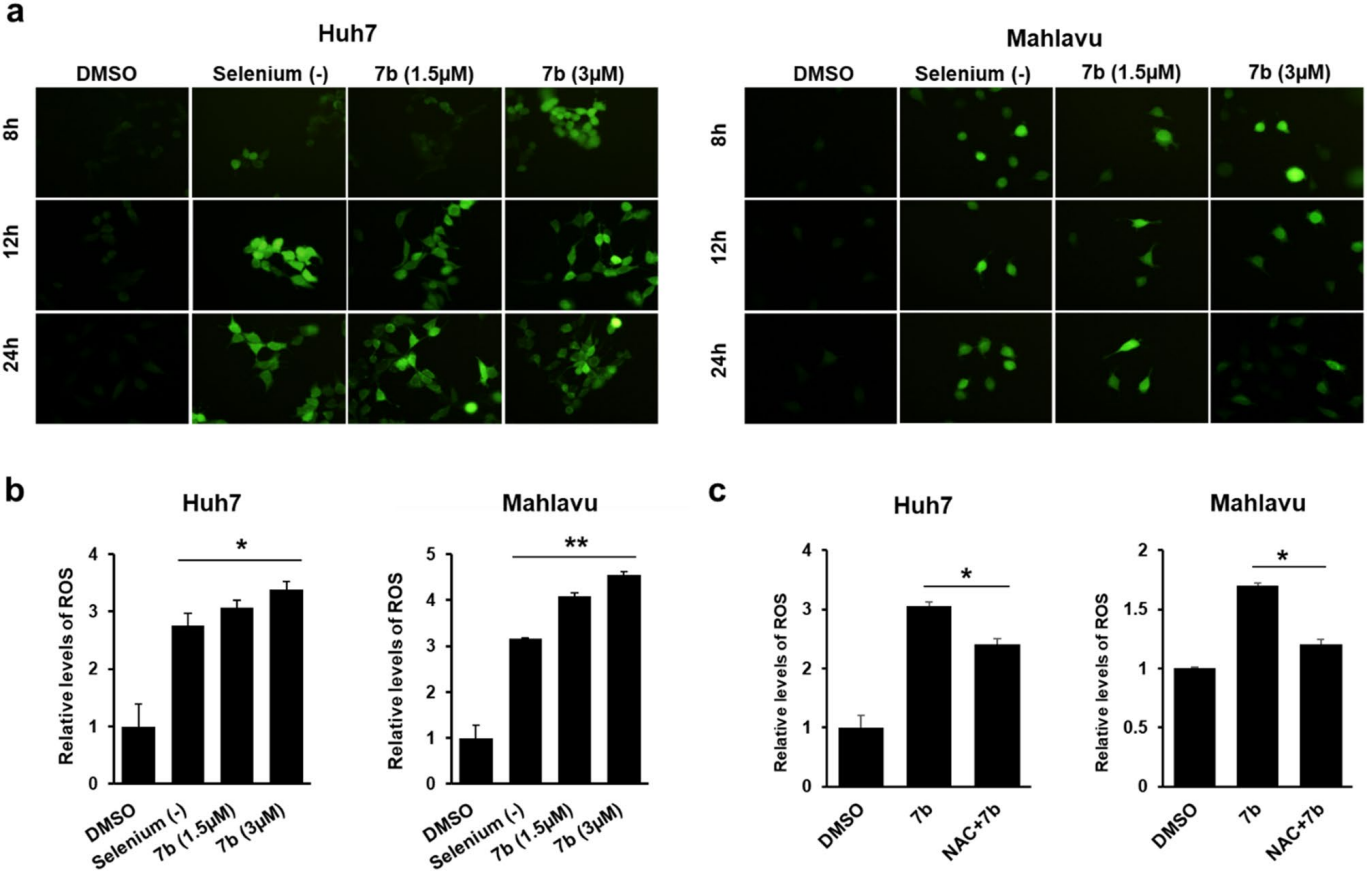
**7b **induces oxidative stress in HCC cell lines. (**a**) Representative images of DCFH-DA staining of Huh7 and Mahlavu cells treated with Se-deficient media or increasing concentrations of **7b** for 8, 12, and 24 h. All images are obtained under a fluorescent microscope with 20 × magnification. (**b**) Flow cytometric analysis of ROS accumulation in HCC cells. Bar graphs indicate the fold change and quantitative analysis of ROS-positive cells relative to DMSO after 12 h for each treatment condition. Se-deficient medium was used as a positive control for ROS induction. (**c**) Quantitative analysis of reversal of ROS induction upon NAC pretreatment in HCC cells is represented in bar graphs. Cells were either treated with 3 µM **7b** only or 5 mM NAC for 1 h and then with **7b** for 12 h. ROS accumulation in HCC cells was analyzed using flow cytometry.

The most common underlying mechanisms inducing G2/M arrest followed by apoptosis in cells are ER-stress and the accumulation of reactive oxygen species (ROS)^38,39^. Since XBP-1 splicing via semi-quantitative PCR is indicative of ER-stress induction^40^, we evaluated the spliced XBP-1 in Huh7 cells upon treatment with compound **7b,** DMSO or 500 ng/mL tunicamycin (TN) for 24 h. Tunicamycin was used as a positive control at an ER stress-inducing dose^41^. Compound **7b** did not induce ER-stress-specific XBP-1 splicing in these cells, unlike TN (Supplementary Figure S3). Then, we tested whether oxidative stress upon ROS accumulation was triggered by compound **7b** in Huh7 and Mahlavu cells, which were treated with increasing concentrations of compound **7b** (1.5 and 3 µM) for 8, 12, and 24 h. In the presence of compound **7b,** the accumulation of ROS was detected significantly in both Huh7 and Mahlavu cells by DCFH-DA staining, which was in parallel quantified and statistically analyzed by flow cytometric analysis (Fig. 4a and b). Additionally, ROS induction was significantly decreased once the cells were pretreated with NAC prior to **7b** treatment (Fig. 4c). NAC pretreatment decreased apoptosis caused by **7b** and resulted in lower levels of cell cycle arrest at the G2/M phase (Suppl. Fig. S4), which further supported the compound's mechanism of action as oxidative stress-induced apoptosis.

**Figure 5:**
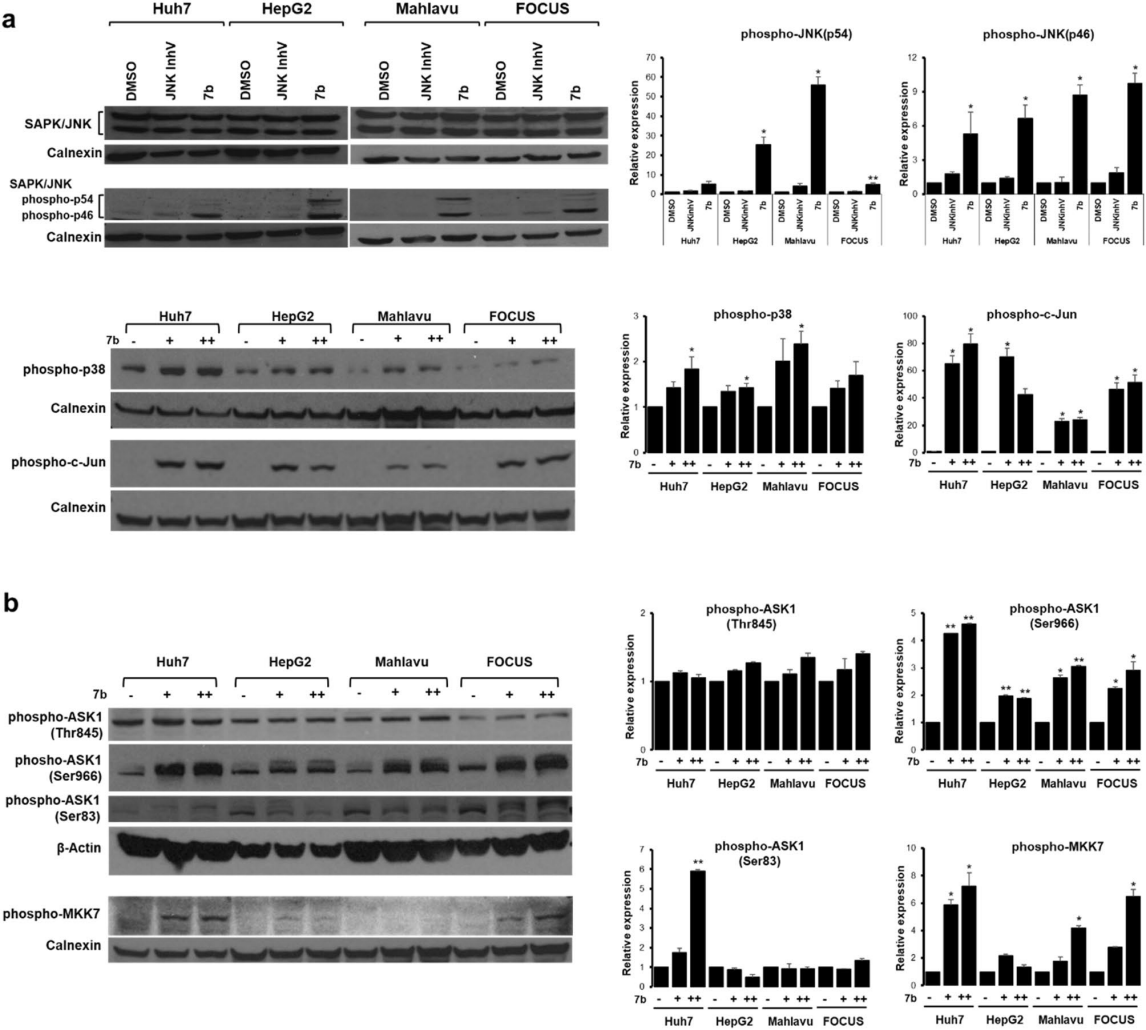
**7b **activates the JNK pathway in HCC cell lines. (**a**) Representative western blot images of total or active (phosphorylated) JNK1 and JNK2 protein levels in HCC cell lines treated with increasing concentrations of compound **7b**, cell line-specific IC_50_ concentrations of JNKInhV (Huh7: 2 μM, HepG2: 2 μM, Mahlavu: 5 μM, FOCUS: 0.5 μM), and DMSO control (top left panel); phospho-p38 and phospho-c-Jun levels in HCC cell lines treated with increasing concentrations of **7b** (+ : IC_50_, +  + : 2 × IC_50_) and DMSO control (bottom left) for 24 h. Bar graphs represent quantitative analysis of the relative band intensity values obtained from 3 different experiments of each treatment group for phosphorylated JNK, phosphorylated c-Jun proteins compared to DMSO using the ImageJ analysis tool (right panel). (**b**) Representative images indicate the changes in the levels of P-MKK7, P-ASK1^Thr845^, P-ASK1^Ser966^ and P-ASK1^Ser83^ proteins by western blot analysis. Huh7, HepG2, Mahlavu, and FOCUS cells were treated with increasing concentrations of compound **7b** and corresponding DMSO for 24 h (bottom left panel). Bar graphs represent quantitative analysis of the relative band intensity values obtained from 3 different experiments of each treatment group for MKK-7, and phosphorylated ASK1 proteins compared to DMSO using ImageJ analysis tool (right panel). Calnexin or β-actin were used as equal loading controls for all western blot experiments.

It is well described in the literature that activation of c-Jun NH2-terminal kinases (JNKs), also known as stress-activated protein kinases (SAPKs), is a typical response to many forms of stress^42,43^. Therefore, we checked the levels of activated JNK and its downstream element c-Jun, a transcription factor phosphorylated by JNK1 and JNK2, in HCC cells treated with **7b**, or JNKInhV relative to DMSO treated cells for 24 h. We observed a significant increase in the phosphorylated-JNK and phosphorylated-c-Jun protein levels upon compound **7b** treatment (Fig. 5a). We tested the effect of **7b** on the regulation of p38, another ROS-mediator-activated protein ^44^, and found that phospho-p38 levels also increase significantly upon **7b** treatment in HCC cell lines (Fig. 5a). To further investigate the effects of ROS accumulation on the upstream components of the JNK signaling pathway, levels of MKK-4, MKK7^45^ and apoptosis signal-regulating kinase 1 (ASK1) proteins^46,47^ were analyzed. We found that compound **7b** increased the phospho-MKK7 protein levels (Fig. 5b) but did not change the phospho-MKK4 levels in HCC cells (Supplementary Fig. S5). Furthermore, **7b** also altered the phosphorylation status of its upstream activator ASK1 (Fig. 5b). In contrast, NAC pretreatment reduced levels of JNK phosphorylation in HCC cells, further supporting our findings that JNK pathway activation is caused by ROS accumulation (Supplementary Fig. S6). To further analyze the role of JNK pathway activation and apoptosis induction by **7b**, HCC cells were treated with JNK inhibitor together with **7b** for 24 h and apoptotic cells were measured by Annexin-V/PI staining. Cells were partially resistant to apoptosis in the presence of JNK inhibitor compared to cells treated with **7b** alone (Supplementary Fig. S4).

### In vivo anti-tumor activity of compound 7b in mouse xenografts

**Figure 6:**
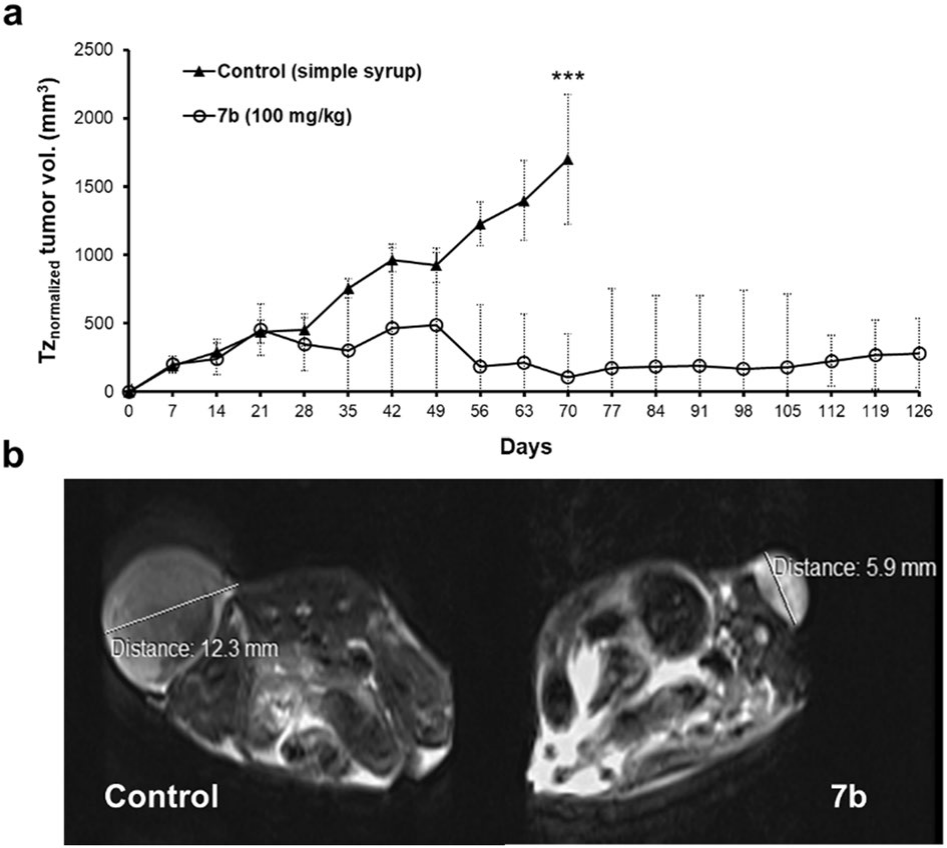
**7b** reduces tumor growth and increases the overall survival of nude mice. (**a**) Mahlavu xenografted nude mice were treated with either **(**100 mg/kg) compound **7b** suspended in simple syrup or only simple syrup twice a week once the tumor size reached a volume of 150 mm^3^. Tumor sizes are recorded twice a week throughout the experiment. Time-zero normalized data are shown as mean ± SD for each group of animals (*n* = 5); ***P < 0.001, as calculated using one-way ANOVA (**b**) Representative MRI images taken at the 135^th^ day for compound **7b** treated group and 105^th^ day for control.

Mahlavu cell xenografts in nude mice were used to determine the in vivo anti-tumor effect of compound **7b**. Mahlavu cells were selected due to their significant response to compound **7b** and their poorly differentiated and highly metastatic nature^48^. We observed that compound **7b** increased the overall survival of nude mice (Fig. 6a and Supplementary. Fig. S7). Moreover, the tumor volumes were significantly decreased in the compound **7b** treated group, as demonstrated by the representative MRI images (Fig. 6b). Altogether, our in vivo data revealed that compound **7b** is orally tolerable in nude mice and is a novel and potentially effective drug candidate against HCC.

### Effect of compound 7b on liver cancer stem cell enrichment

**Figure 7:**
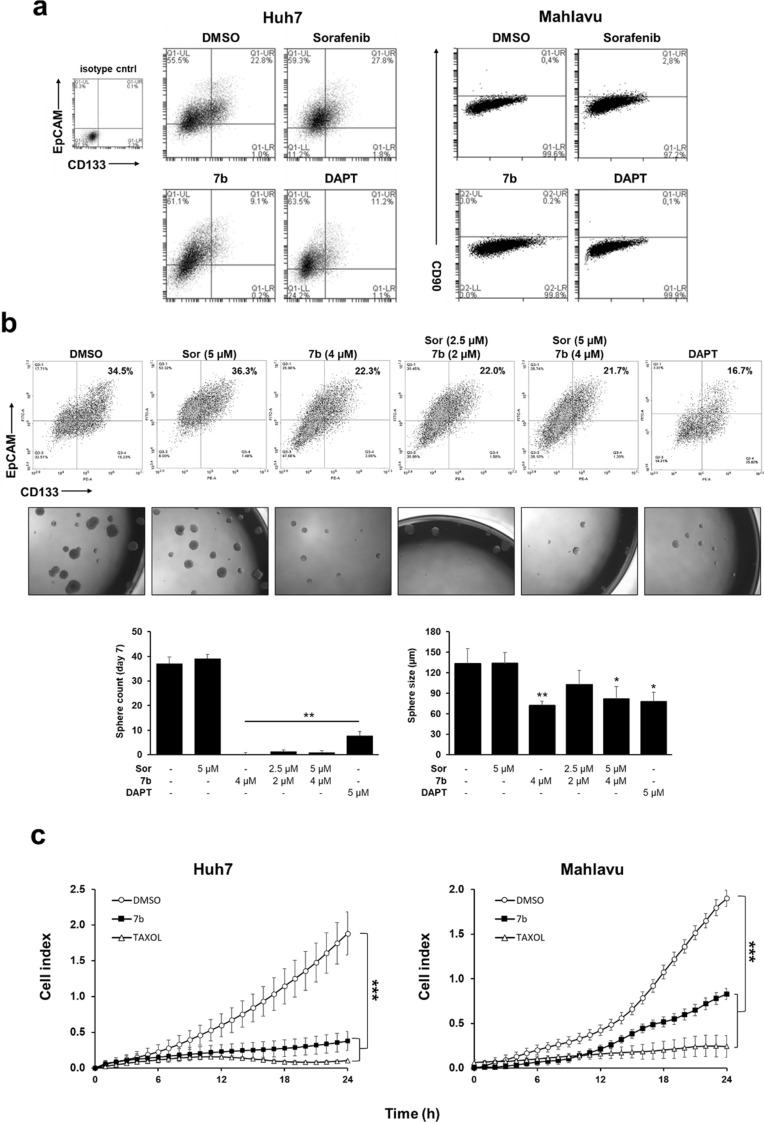
**7b **reduces enrichment of LCSCs and the migration capacity of HCC cells**.** Huh7 and Mahlavu cells were treated with sorafenib (5 µM), DAPT (5 µM), compound **7b** (2 µM), or DMSO control for 72 h. (**a**) Images represent flow cytometric analysis of CD133/EpCAM positivity in Huh7 cells, and CD90 positivity in Mahlavu cells upon treatment with indicated compounds/inhibitors. Upper-right quadrant (UR): double-positive cells for CD133 and EpCAM for Huh7 cells; upper-window: positivity for CD90 for Mahlavu cells. (**b**) Flow cytometric analysis of CD133/EpCAM positivity in Huh7 cells treated with compound **7b** or sorafenib alone and with combinations of compound **7b** and sorafenib and representative images of spheres formed by Huh7 cells. Bar graphs indicate quantitative analysis of sphere count and diameter in different treatment groups. (**c**) Cell migration index of Huh7 and Mahlavu cells treated with the positive control, taxol (20 ng/ml) or compound **7b** (2 µM for Huh7 and 3 µM for Mahlavu) for 24 h.

Tumor initiating cells of liver, so-called (liver cancer stem cells-LCSCs), are known to be capable of reconstituting the tumor by themselves, triggering metastatic events, and enhancing drug resistance in cancer cells^49^. Therefore, we tested compound **7b** for its effect on enrichment LCSCs, which can be quantified by detecting CSC markers found on the surface of these cells. For this purpose, Huh7 and Mahlavu cells were treated with compound **7b** and other inhibitors such as sorafenib, DAPT, or DMSO control at IC_50_ concentrations for 72 h. Cells that remained viable after treatment were collected, and the expression of LCSC markers CD133 and EpCAM (for Huh7) and CD90 (for Mahlavu) were evaluated by flow cytometry. Results have revealed that compound **7b** was able to decrease the CD133 + /EpCAM + population in Huh7 cells and the CD90 + population in Mahlavu cells significantly (Fig. 7a). Besides, sorafenib was known to enrich the LCSC population, as demonstrated previously^36^. To test the combinatory effect of compound **7b** with sorafenib, Huh7 cells were treated with the increasing concentrations of both compounds simultaneously for 72 h. Flow cytometry analysis and the sphere formation assay have shown that compound **7b** alone and, in combination with sorafenib, reduced the LCSC ratio and the sphere formation capacity of Huh7 cells. (Fig. 7b).

### Effect of compound 7b on cell migration

To identify the effect of compound **7b** on the migration capacity of HCC cells, a real-time cell migration system (RT-CES, DP system, xCELLigence) was used. Huh7 and Mahlavu cells were treated with taxol as a positive control for cell migration inhibition or with compound **7b** (Huh7: 2 µM, Mahlavu: 3 µM) for 24 h. Comparable with the effect of taxol, compound **7b** inhibited the migration of both cell lines compared to the DMSO treated cells (Fig. 7c). Altogether, **7b** could effectively interfere with the drug resistance-related cellular mechanisms such as stemness and migration.

## Discussion

HCC is one of the most common and deadly cancers in the world. Sorafenib, which is a multi-kinase inhibitor approved by FDA was reported to prolong survival of advanced stage HCC patients by only 3 months^50^. Although second-line treatment options such as Regorafenib were also studied for patients who are intolerant to sorafenib, drug resistance has become a critical problem for these patients due to the highly heterogeneous molecular nature of HCC.

Recent studies have reported that NSAIDs effectively decrease the incidence and mortality of various types of cancers^12,51,52^. In this study, we demonstrated that compound **7b**, a synthetic 1,2,4-triazolo[3,4-b]-1,3,4-thiadiazine NSAID derivative, is a potent anticancer agent for HCC cells in vitro and in vivo, which was previously reported to have promising analgesic and anti-inflammatory activities^25,29^. Among the reported numerous biological effects, accumulation of reactive oxygen species (ROS) is considered as the underlying mechanism of the anticancer potential of NSAIDs^53,54^. Compatible with the literature, we have demonstrated that ROS accumulation induces growth inhibition, apoptosis, and G2/M cell cycle arrest in the presence of **7b** (Figs. 3 and 4). We also examined signaling pathways activated by oxidative stress and found that activation of JNK and p38 as well as the downstream protein c-Jun was induced upon **7b** treatment in HCC cells. (Fig. 5a). Further analysis of the upstream components of the JNK pathway revealed that compound **7b** treatment causes cell line-specific activation of MKK7 protein (Fig. 5b) as well as dynamic regulation of phosphorylation sites on the ASK1 protein (Fig. 5b). Our in vivo data supported our findings on anticancer activity of **7b,** where orally administered compound increased the overall disease-free survival for more than 4 months and reduced tumor size significantly in Mahlavu xenografts in nude mice (Fig. 6).

One of the most important factors associated with drug resistance in HCC is the presence of cancer stem cells. Conventional therapies fail to affect slowly dividing stem cell-like cancer cells and mainly target rapidly dividing tumor cells^55^. CSCs can reconstitute the tumor by themselves and manage to acquire metastatic features to migrate to distant organs^56,57^. Hence, we have also tested the compounds against LCSC and revealed that compound **7b** was also effective in inhibiting the enrichment of LCSCs (Fig. 7), meaning that compound **7b** is not solely active on tumor cells (non-stem), but also tumor-initiating cells in HCC. We also tested the effect of compound **7b** on the migration capacity of HCC cells, since CSC activity is associated with the migration and metastatic capacity of cancer cells^58^. **7b** could effectively inhibit the migration of Huh7 and Mahlavu cells, comparable to the effect of Taxol, which is known as a microtubule stabilizing agent^59^ that is widely used as a positive control for inhibition of migration in cancer cells^60,61^.

In the last decade, the anticancer and anti-metastatic effects of a well-defined NSAID, aspirin, has been described in cancer including HCC^62^. It is reported that by the induction of metabolic and oxidative stress in HepG2 cells, aspirin causes apoptosis through mitochondrial dysfunction^63^. In another study, aspirin was shown to attenuate pro-metastasis caused by sorafenib by upregulating the tumor suppressor HTATIP2 in nude mice xenografts^64^. Yet, the use of aspirin in treatment of cancer and its prevention remains uncertain due to the problems with the optimal dosage of aspirin and its serious side-effects such as gastrointestinal bleeding, increased uric acid, and coagulation inhibition^65,66^. Since these drugs are not designed to treat or prevent cancer progression, designing and developing novel NSAIDs that target cancer-related mechanisms is very crucial and can be considered as a new class of anticancer pharmaceutical agents such as non-steroid anti-inflammatory chemotherapeutic drugs (NSAICD).

**Figure 8:**
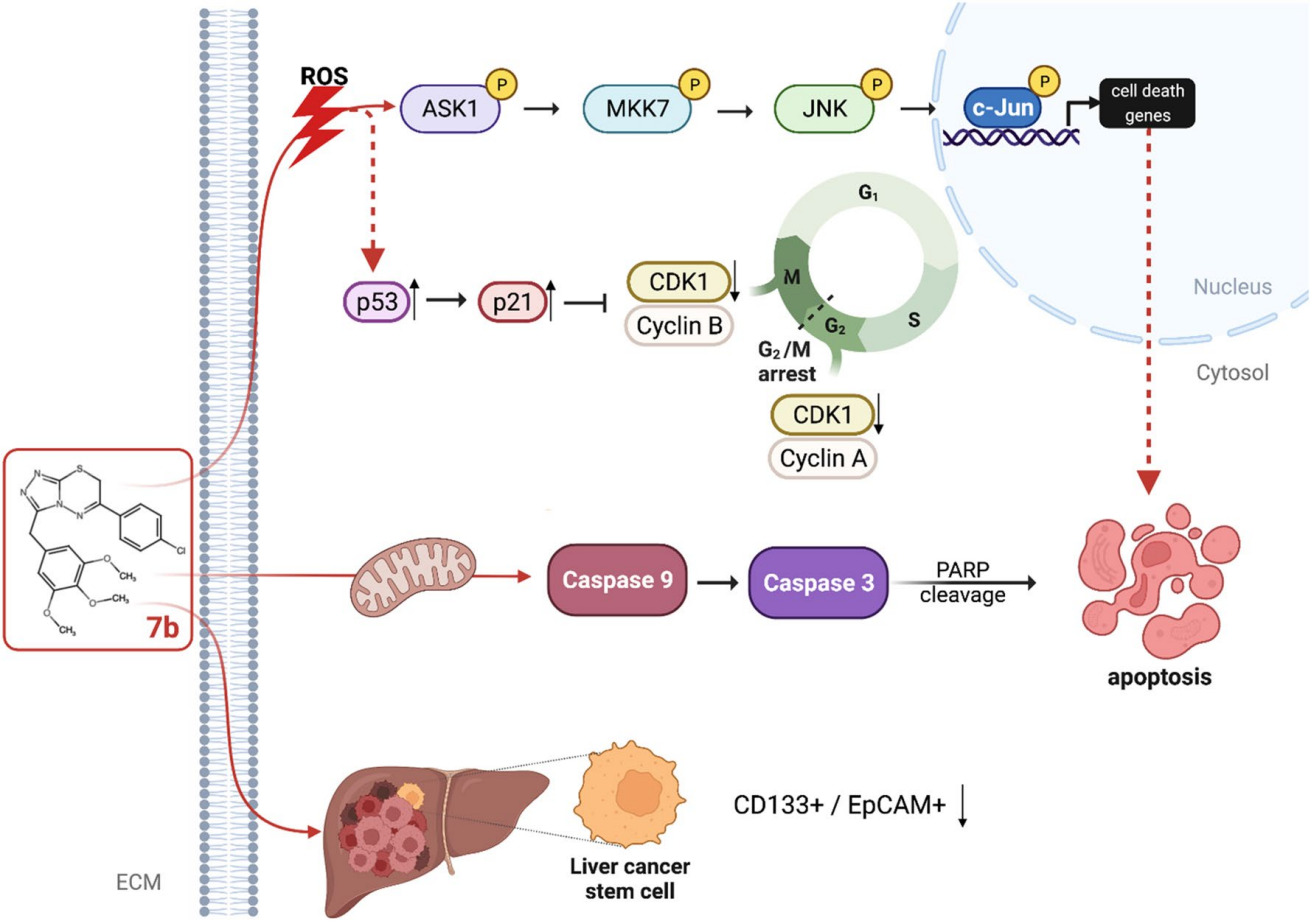
Illustration of molecular mechanisms involved in response to compound **7b** treatment in HCC cell lines. **7b** blocks cell proliferation through induction of ROS accumulation and JNK pathway activation which further stimulates transcription of genes related to apoptosis and cell cycle arrest. As a result, G2/M arrest (through the regulatory effect of p53 and p21), and apoptosis (through intrinsic pathway) are observed. **7b** is also a potent compound acting on cancer stemness and migration capacity of HCC cell lines. This figure was created with BioRender.com.

In conclusion, our results revealed that **7b**, a synthetic 1,2,4-triazolo[3,4-b]-1,3,4-thiadiazine NSAID derivative, is a promising compound with anticancer and anti-stem cell activities against HCC cells in vitro and in vivo. The anticancer effect was attributed to the induction of oxidative stress, cell cycle arrest, and eventually apoptosis through the JNK pathway regulation (Fig. 8). These findings highlight the potency of **7b** as a new NSAICD, bearing a thiadiazine core, to be considered a promising anticancer drug for HCC patients and deserves further analysis.

## Supplementary Information

Supplementary Information.

## Abbreviations

HCC: Hepatocellular carcinoma
NSAID: Nonsteroidal anti-inflammatory drug
ROS: Reactive oxygen species
SRB: Sulforhodamine B
DMSO: Dimethyl sulfoxide
LCSC: Liver cancer stem cell
RT: Room temperature
IC50: 50% Inhibitory concentration
DMEM: Dulbecco's Modified Eagle Medium
FBS: Fetal bovine serum
PBS: Phosphate-buffered saline
CI: Cell index
RT-PCR: Real-time polymerase chain reaction
Se: Selenium
DCFH-DA: Dichloro-dihydro-fluorescein diacetate
