# Supplementary material for: A new triazolothiadiazine derivative inhibits stemness and induces cell death in HCC by oxidative stress dependent JNK pathway activation
Source: Sci Rep. 2022 Sep 7;12:15139. doi: 10.1038/s41598-022-17444-0 (PMC9452548; doi:10.1038/s41598-022-17444-0)
Supplement: Supplementary file 1 — Supplementary Information. [file 41598_2022_17444_MOESM1_ESM.pdf]

A new triazolothiadiazine derivative inhibits stemness and induces cell death in HCC by oxidative stress dependent JNK pathway activation

Deniz Cansen Kahraman[1, 6], Ebru Bilget Guven[2, 3, 6], Peri S. Aytac[4, 6], Gamze Aykut[2], Birsen Tozkoparan[4], Rengul Cetin Atalay[5]

Supplementary Information

Supplementary Table S1. IC_{50} values of selected compounds **7a**, **7b,** and **7c** in µM concentrations on Huh7, HepG2, Hep3B, PLC, SK-Hep1, Mahlavu, FOCUS, SNU182 and SNU475 cells determined by SRB assay after 72h of treatment.

Compounds	Huh7	HepG2	Hep3B	PLC	SK-Hep1	Mahlavu	FOCUS	SNU182	SNU475
**7a**	8.6	5.6	14.8	23.6	24.3	21.3	13.7	12.4	15.0
**7b**	0.3	0.2	0.8	1.6	6.0	1.0	0.6	0.9	0.5
**7c**	16.4	7.9	30.3	NI	NI	NI	NI	15.4	26.2

* NI: no inhibition

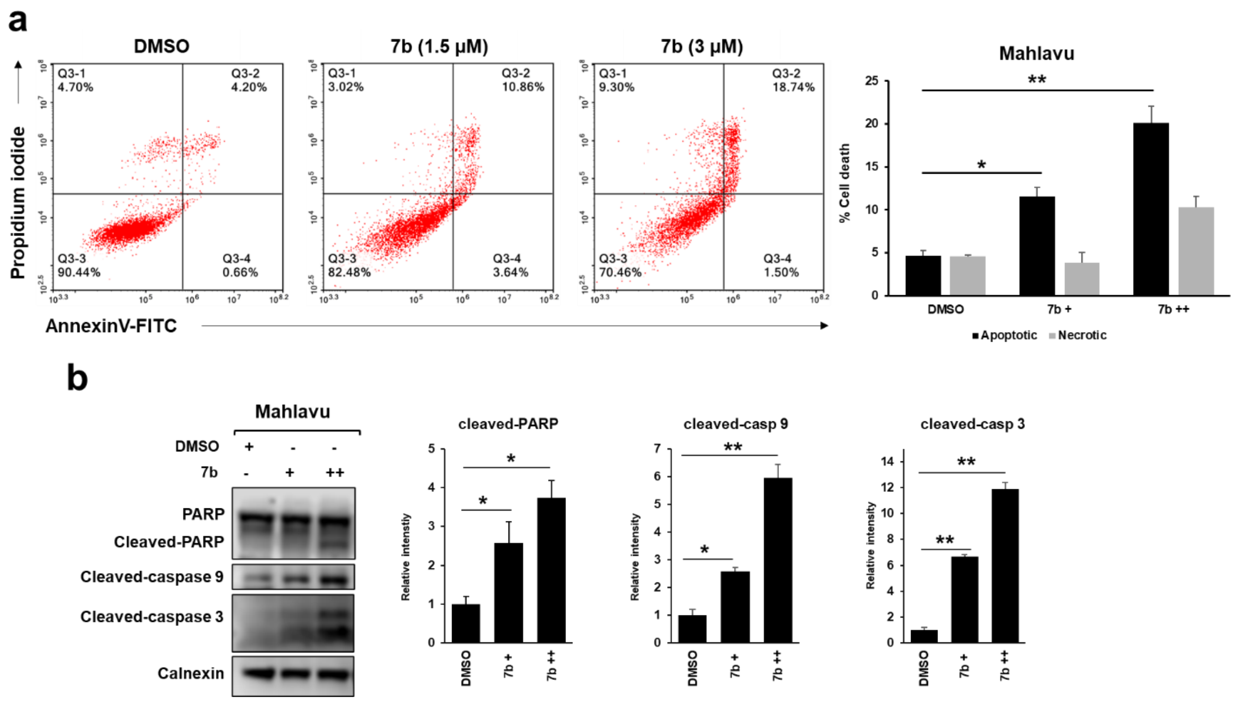

Supplementary Figure S1. 7b induces apoptosis in HCC cells. **a)** Annexin-V/PI staining results of Mahlavu cells treated with indicated concentrations of **7b** for 24h. **b)** Western blot analysis of PARP, cleaved caspase-3, and cleaved caspase-9 in Mahlavu cells treated with increasing concentrations (1.5 and 3 µM) of **7b** after 24h. Relative intensity ratios and statistical significance for each protein are represented in bar graphs. Calnexin was used as an equal loading control.

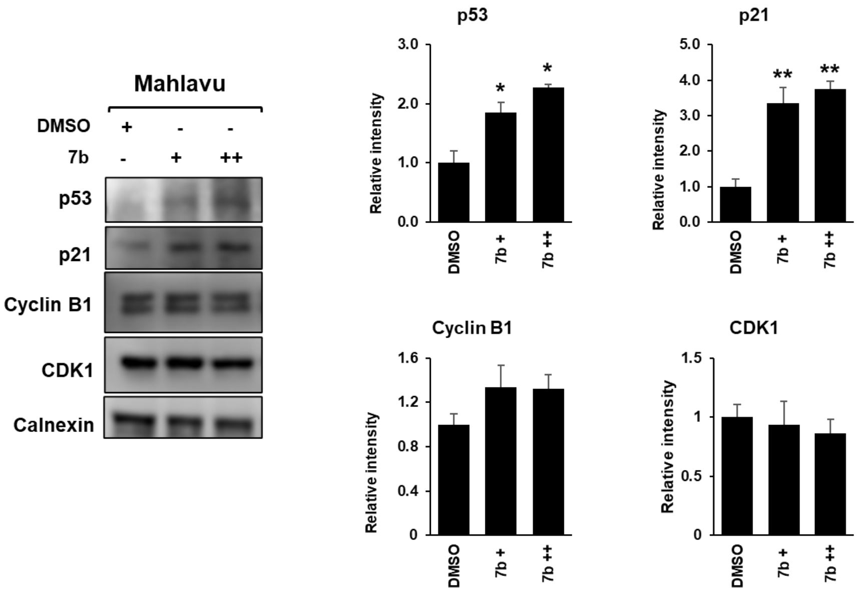

Supplementary Figure S2. 7b induces cell cycle arrest in HCC cells. a) Western blot analysis of G1 arrest associated proteins, p53, p21, Cyclin B1 and CDK1 in Mahlavu cells treated with increasing concentrations of **7b** (1.5 and 3 μM) after 24h. Relative intensity ratios and statistical significance for each protein is represented in bar graphs. Calnexin was used as equal loading control.

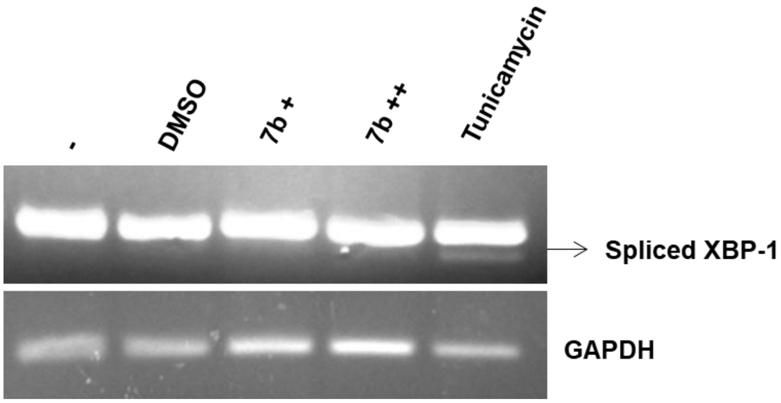

Supplementary Figure S3. PCR analysis of XBP-1 expression in Huh7 cells, treated with increasing concentrations of compound **7b** (-: untreated, DMSO: vehicle control, and 500 ng/mL Tunicamycin: positive control). Spliced XBP-1 is a marker for ER stress.

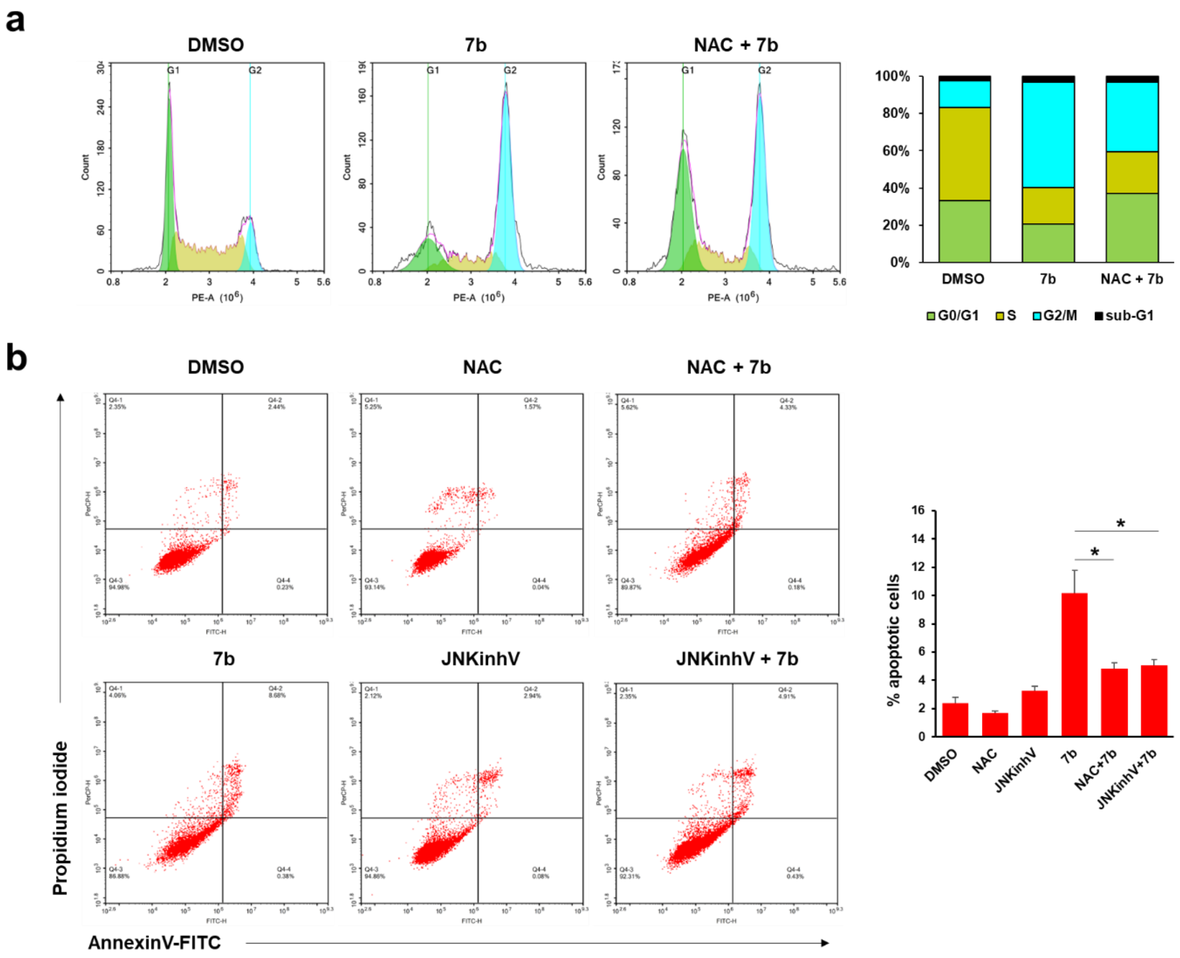

Supplementary Figure S4. NAC decreases cell cycle arrest and apoptosis caused by **7b** and JNK inhibitor reduces **7b** induced apoptosis in Mahlavu cells. **a)** Cells were treated with 1.5 µM **7b** or pre-treated with 5 mM NAC for 1h and then with **7b** for 24h. Cells were collected for PI staining and analyzed by flow cytometry. **b)** For apoptotic cell detection, cells were treated either with 1.5 µM **7b** alone, 1 µM JNKinhV alone, 5 mM NAC alone or with 1 µM JNKinhV+1.5 µM 7b or pre-treated with 5 mM NAC for 1h and then with **7b** for 24h. Cells were collected for Annexin-V/PI staining and analyzed by flow cytometry.

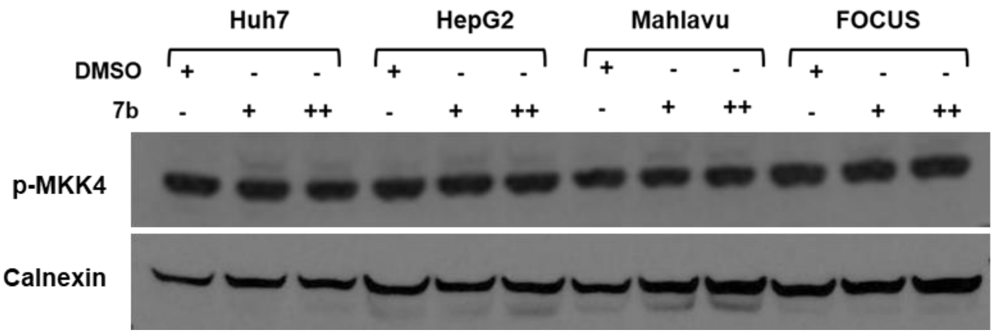

Supplementary Figure S5. The effect of compound **7b** on the protein expression of phospho-MKK4 in HCC cells treated with increasing concentrations of compound **7b** for 24h was determined by western blot analysis. Calnexin is used as an equal loading control.

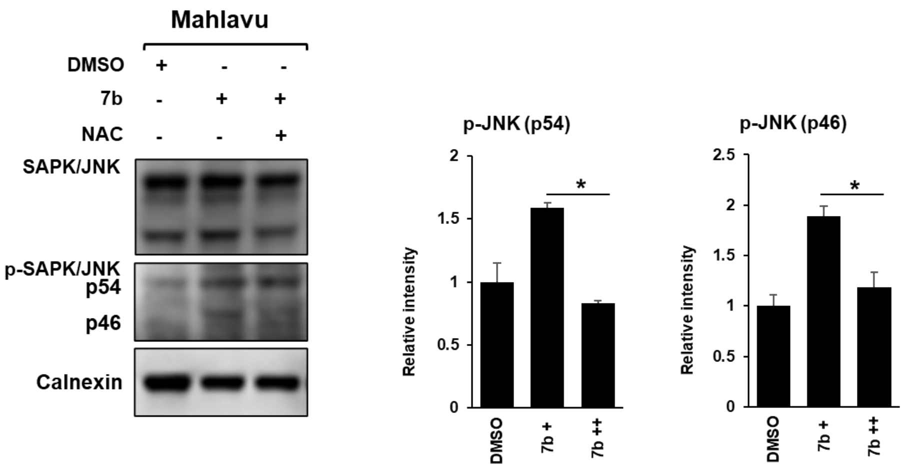

Supplementary Figure S6. NAC pre-treatment reduces JNK phosphorylation induced by **7b**. Western blot analysis of Mahlavu cells treated with **7b** (1.5 µM) or pretreated with NAC (5 mM) and then with **7b** (1.5 µM) for 24h. Relative intensity ratios and statistical significance for each protein are represented in bar graphs. Calnexin was used as an equal loading control.

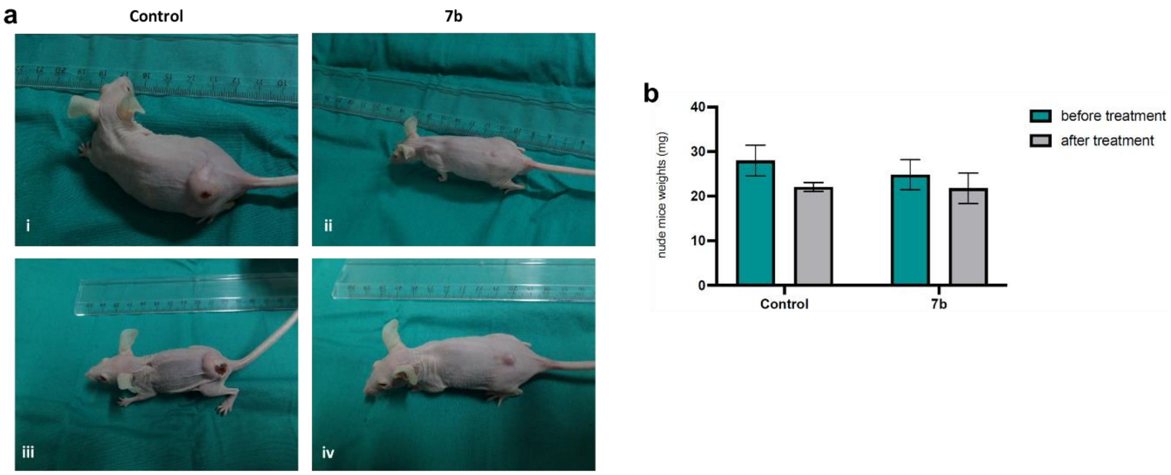

Supplementary Figure S7 **a)** Representative images of animals bearing xenograft tumor for control (i, iii) and 7b (ii, iv) treated groups on their 70th (i, ii) and 100th day (iii, iv) after drug treatment had started. **b)** Recorded measurements during drug treatment displays that the oral administration of compound **7b** does not accelerate body weight reduction compared to the control group.

Fig.3b

PARP

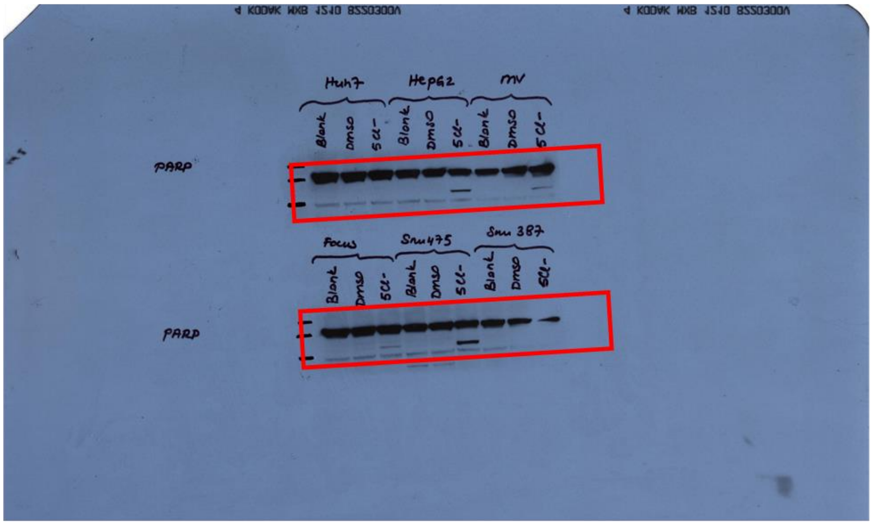

Calnexin of PARP

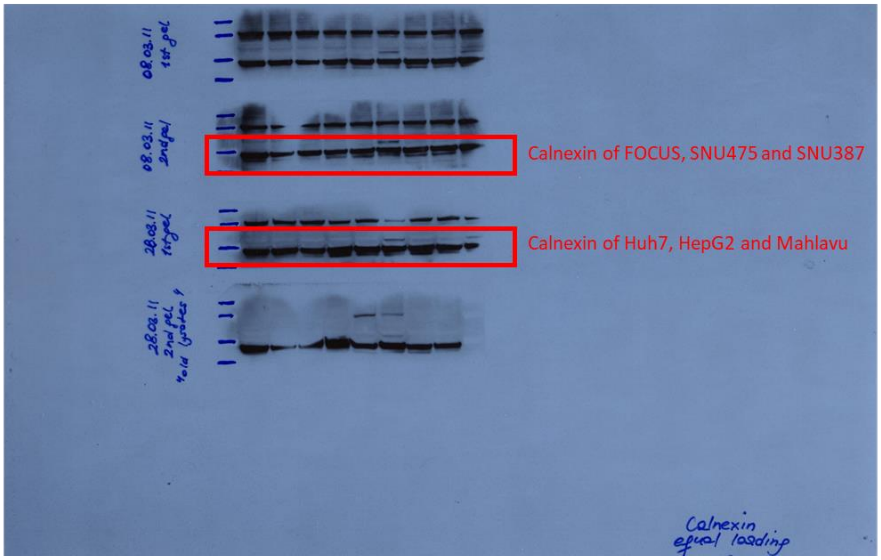

Fig.5a

SAPK/JNK and p-SAPK/JNK of Huh7 and HepG2

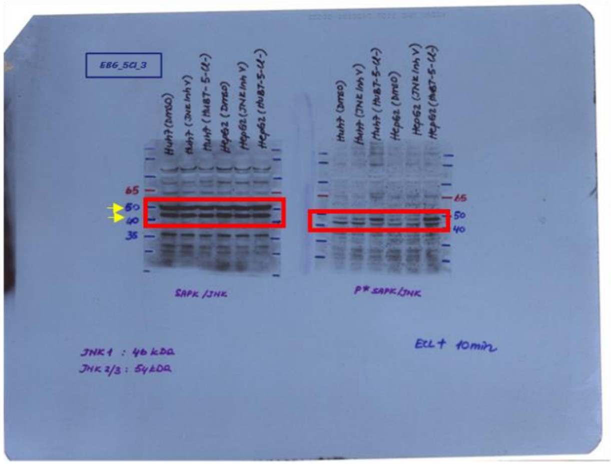

SAPK/JNK and p-SAPK/JNK of Mahlavu and FOCUS

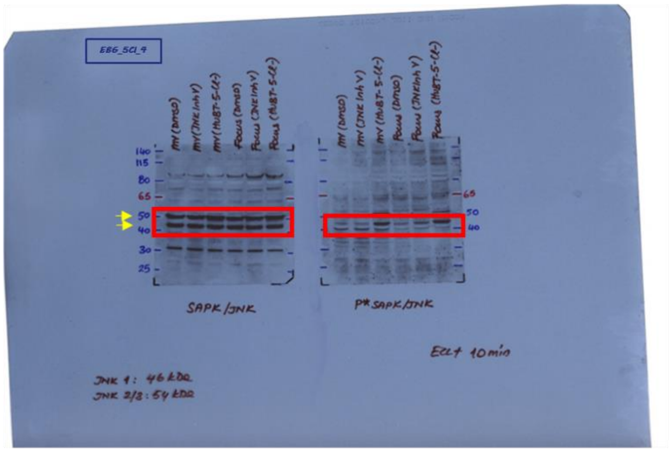

Calnexin of Huh7 and HepG2

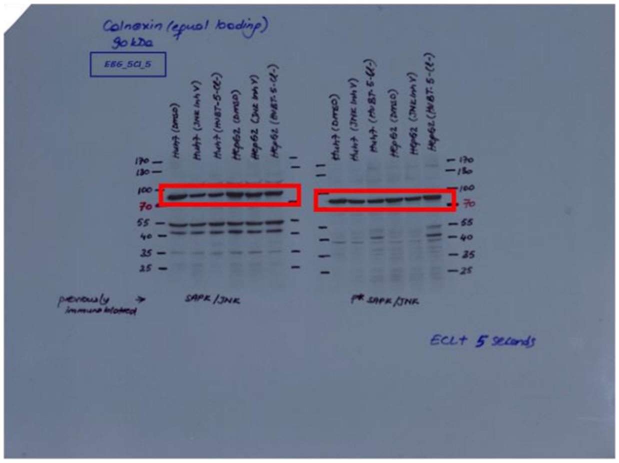

Calnexin of Mahlavu and FOCUS

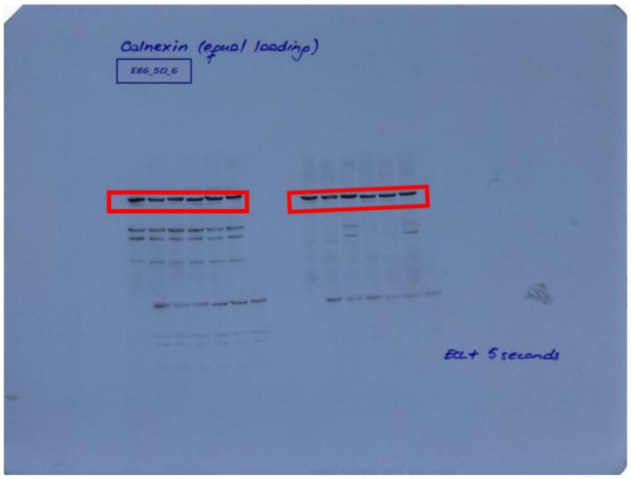

Fig.5a

phospho-p38 and calnexin

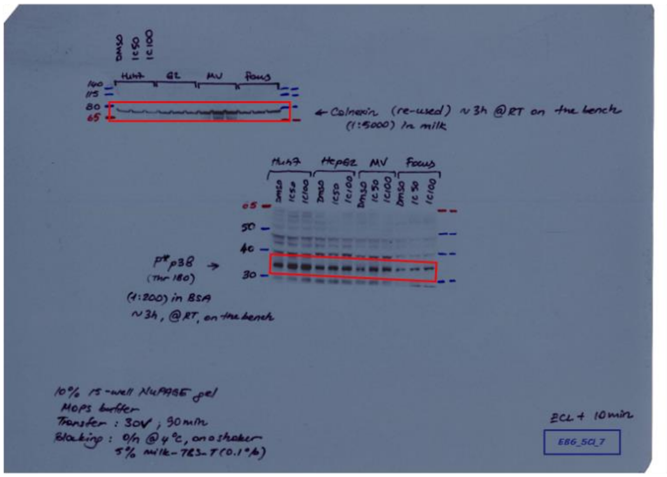

phospho-c-Jun

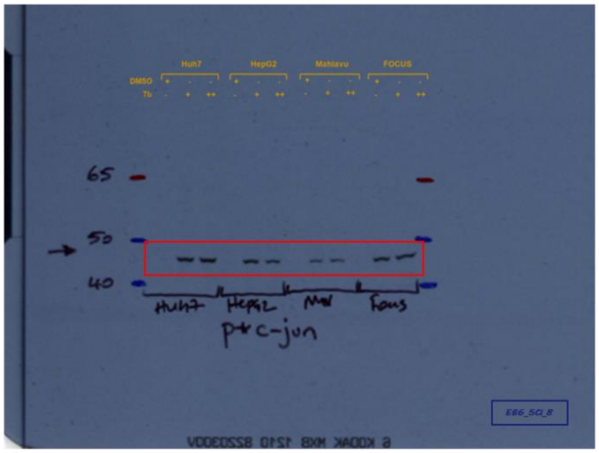

Calnexin of p-c-Jun

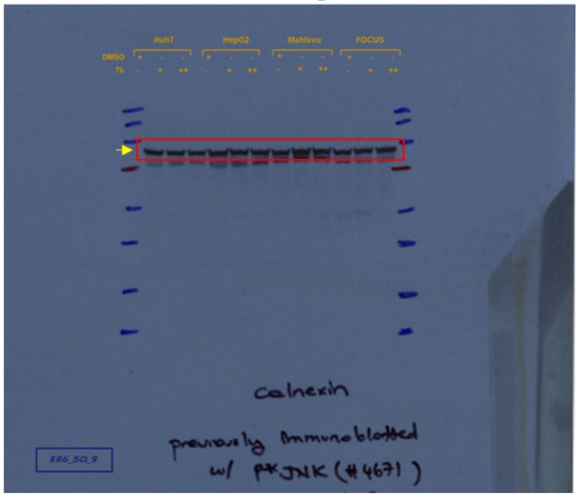

Fig.5b

phospho-ASK (Thr845)

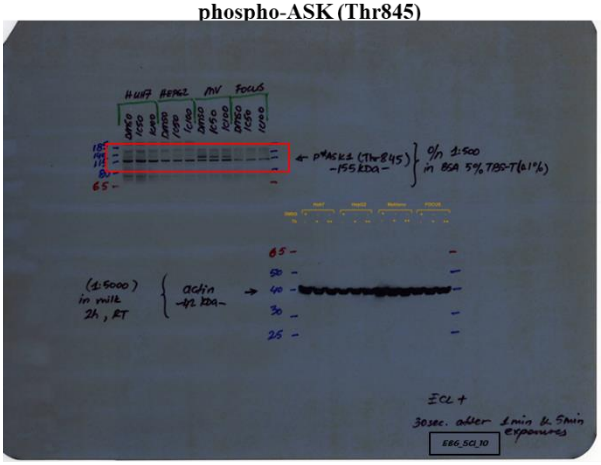

phospho-ASK1 (Ser966) and β-actin

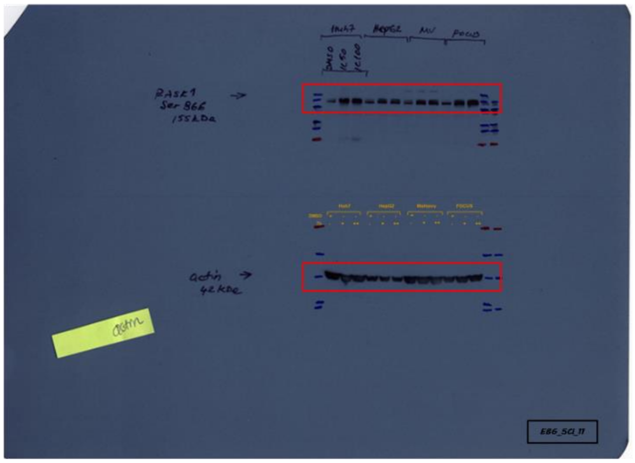

phospho-ASK1 (Ser83)

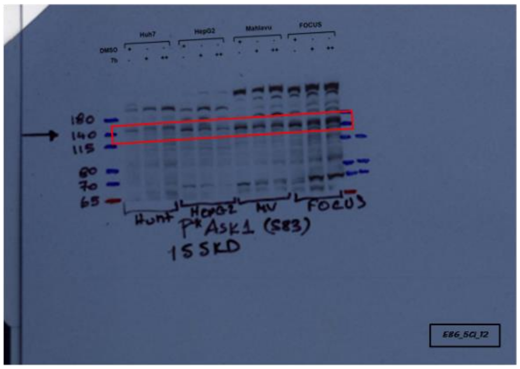

Fig.5b

phospho-MKK7

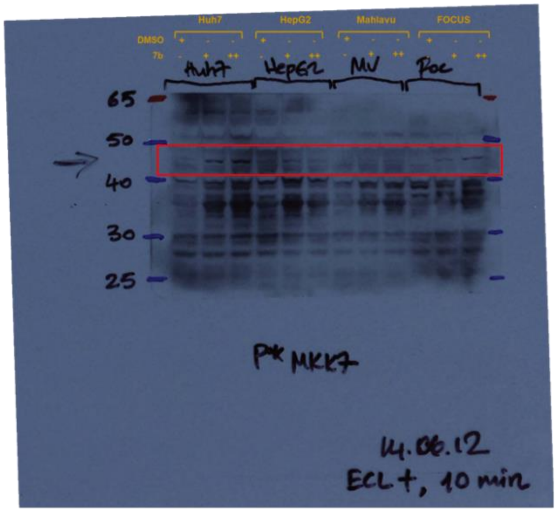

Calnexin of p-MKK7 and p-MKK4

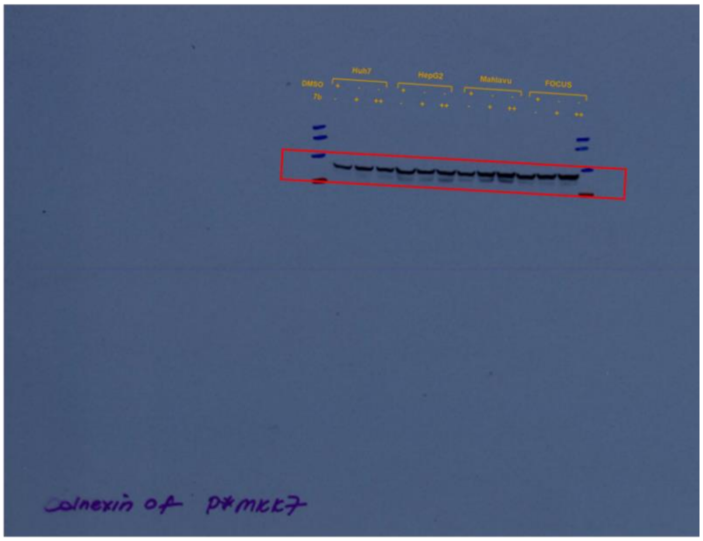

Suppl. Fig. S5

phospho-MEK4/MKK4

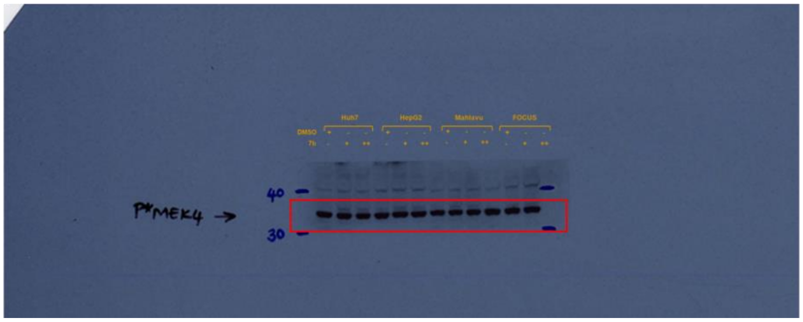

Suppl. Fig. S1b

PARP

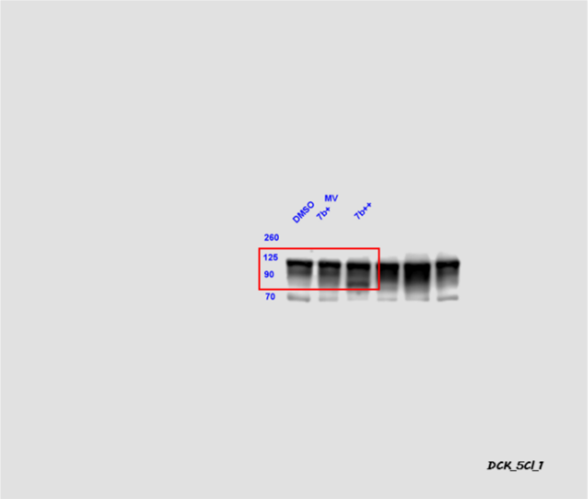

Cleaved casp-9

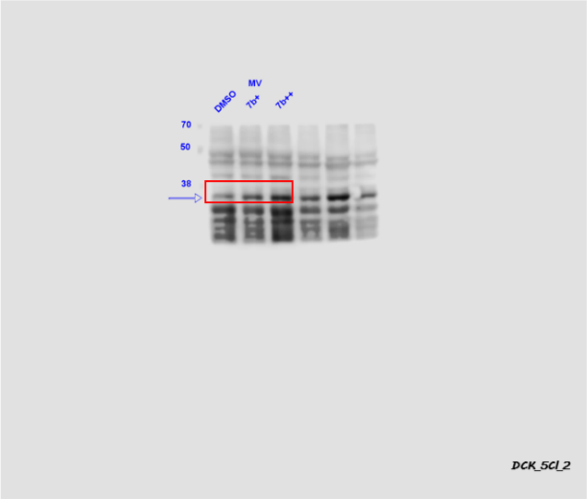

Cleaved casp-3

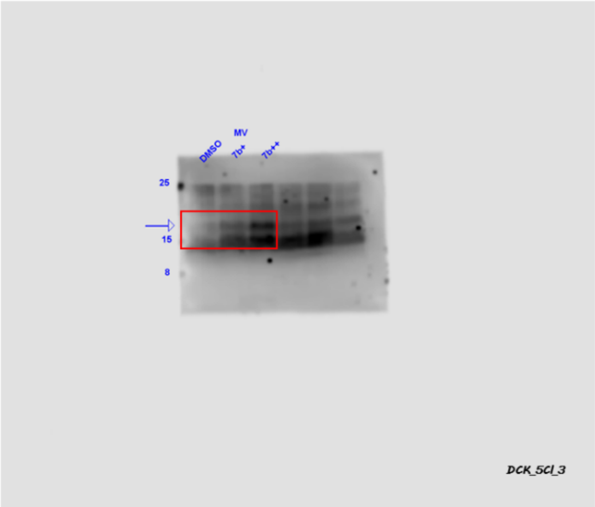

Calnexin

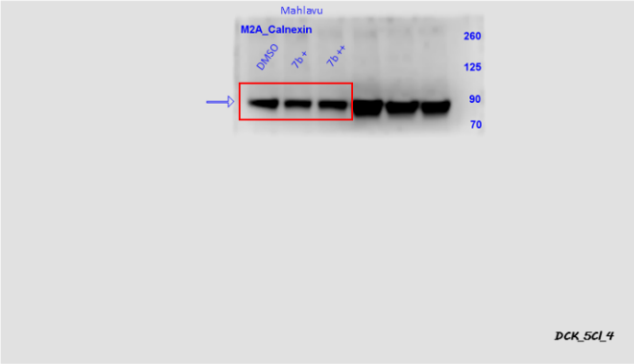

Suppl. Fig. S2

p53

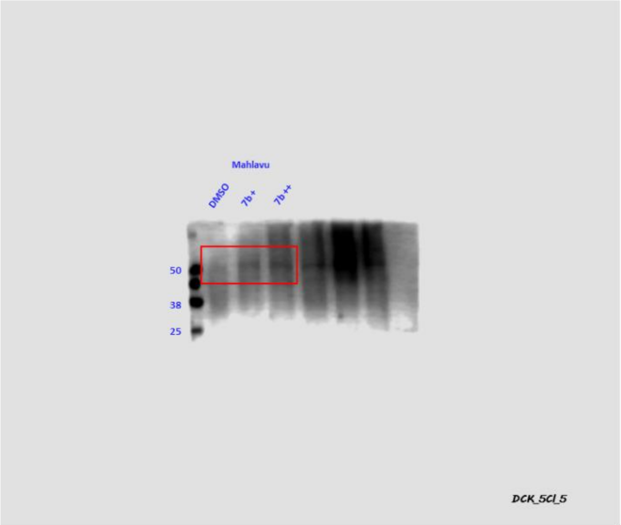

p21

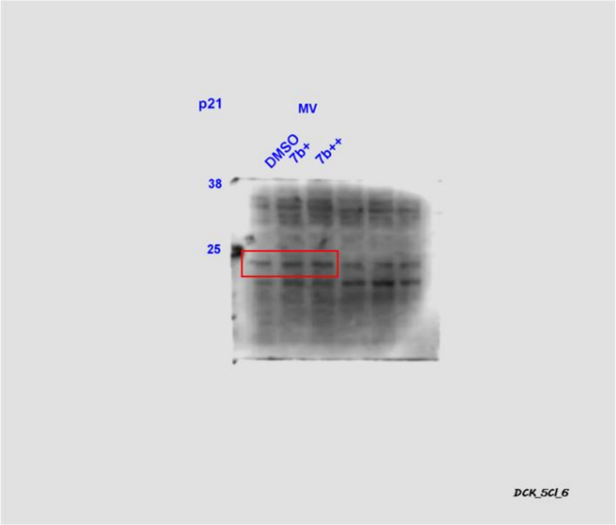

CDK1

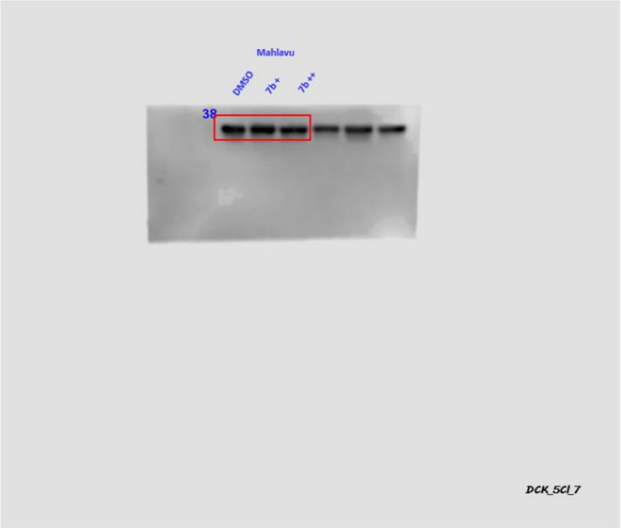

Cyclin B1

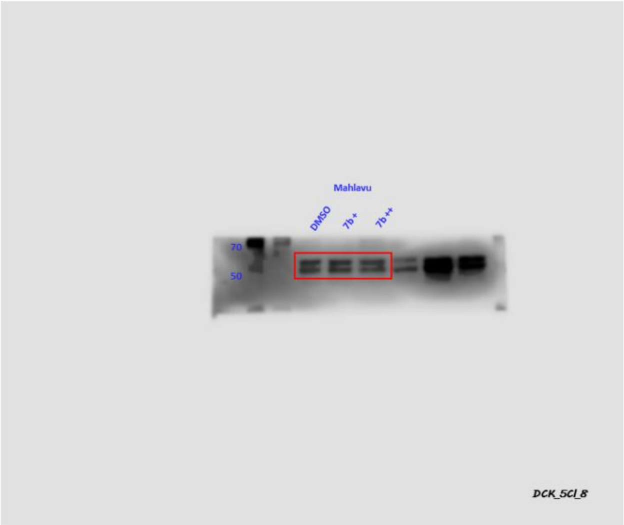

Calnexin

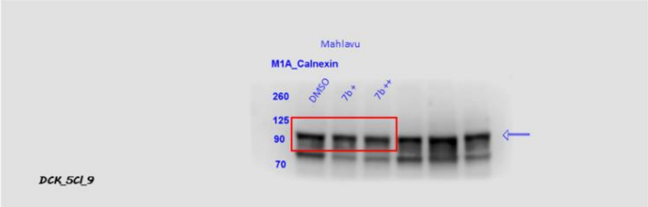

Suppl. Fig. S3

XBP-1

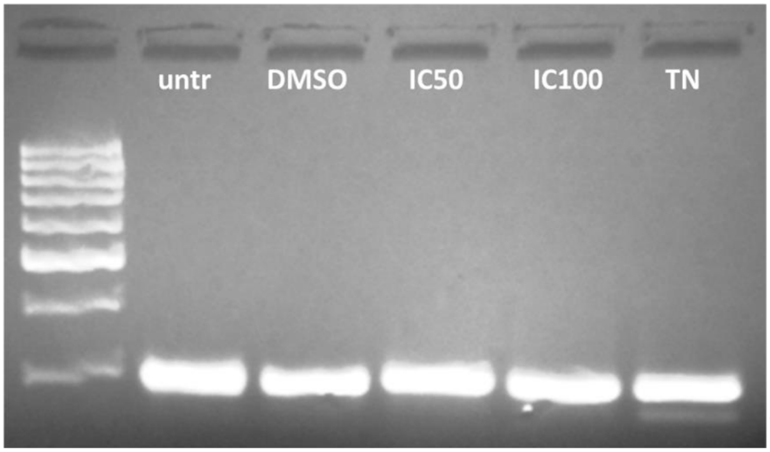

Spliced XBP-1

GAPDH

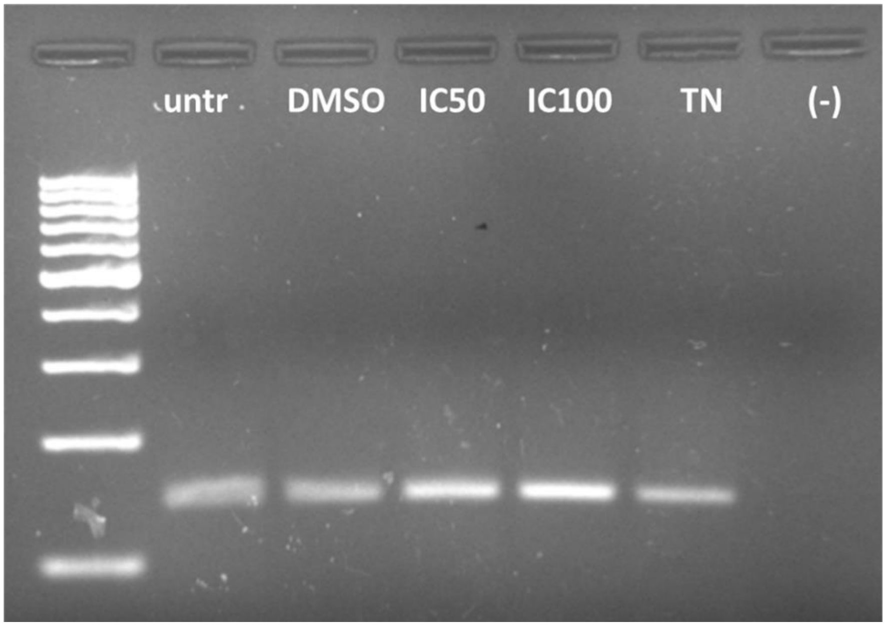

Suppl. Fig. S6

JNK

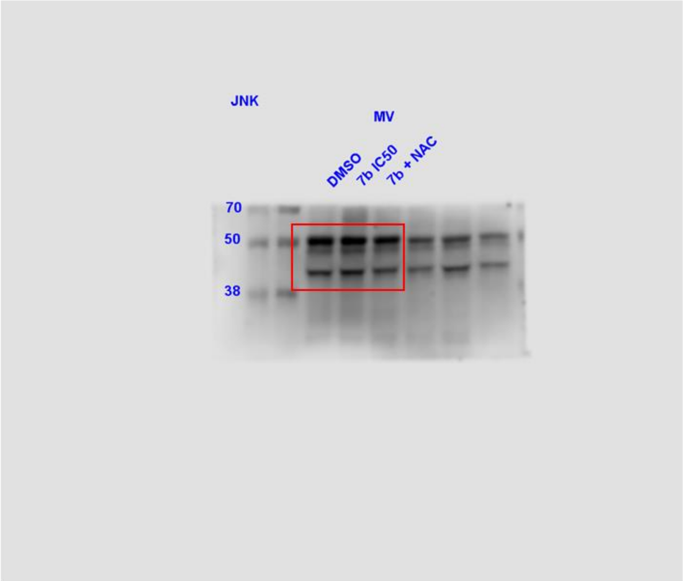

Phospho-JNK

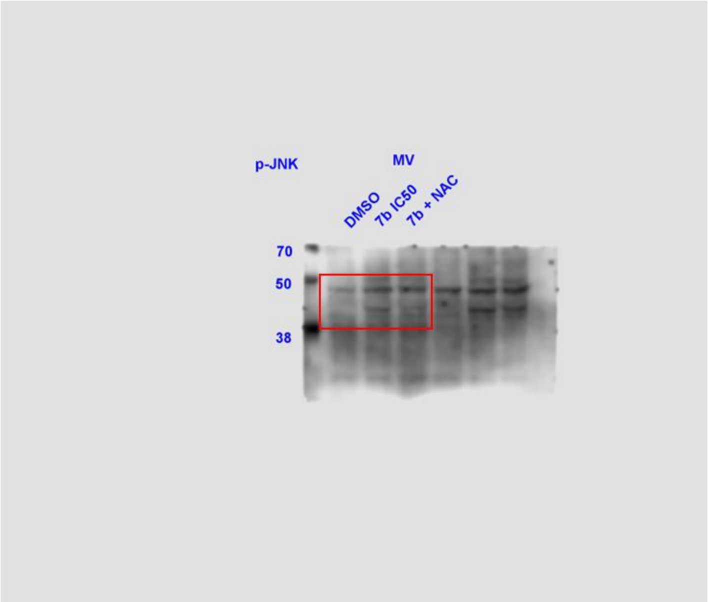

Calnexin

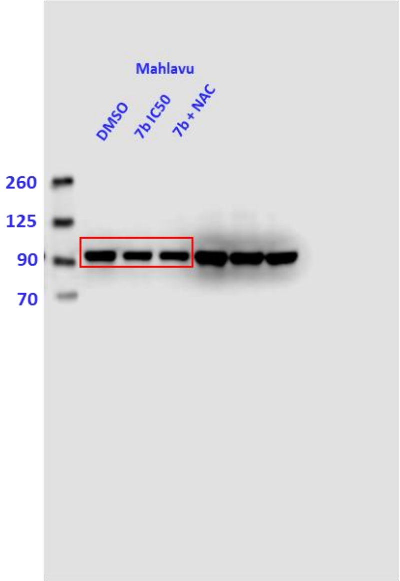

Supplementary Figure S8. Uncropped images of all western blot and gel images represented in the manuscript.
